# Nanomedicines Reprogram Synovial Macrophages by Scavenging Nitric Oxide and Silencing CA9 in Progressive Osteoarthritis

**DOI:** 10.1002/advs.202207490

**Published:** 2023-02-07

**Authors:** Yi Yan, An Lu, Yun Dou, Zhen Zhang, Xiang‐Yu Wang, Lin Zhai, Li‐Ya Ai, Ming‐Ze Du, Lin‐Xia Jiang, Yuan‐Jun Zhu, Yu‐Jie Shi, Xiao‐Yan Liu, Dong Jiang, Jian‐Cheng Wang

**Affiliations:** ^1^ Beijing Key Laboratory of Molecular Pharmaceutics and New Drug Delivery Systems State Key Laboratory of Natural and Biomimetic Drugs School of Pharmaceutical Sciences Peking University Beijing 100191 China; ^2^ Department of Sports Medicine Peking University Third Hospital Beijing 100191 China; ^3^ Laboratory of Innovative Formulations and Pharmaceutical Excipients Ningbo Institute of Marine Medicine Peking University Beijing 315832 China

**Keywords:** carbonic anhydrase IX, cartilage protection, macrophages, nitric oxide, osteoarthritis, polarization, siRNA delivery

## Abstract

Osteoarthritis (OA) is a progressive joint disease characterized by inflammation and cartilage destruction, and its progression is closely related to imbalances in the M1/M2 synovial macrophages. A two‐pronged strategy for the regulation of intracellular/extracellular nitric oxide (NO) and hydrogen protons for reprogramming M1/M2 synovial macrophages is proposed. The combination of carbonic anhydrase IX (CA9) siRNA and NO scavenger in “two‐in‐one” nanocarriers (NAHA‐CaP/siRNA nanoparticles) is developed for progressive OA therapy by scavenging NO and inhibiting CA9 expression in synovial macrophages. In vitro experiments demonstrate that these NPs can significantly scavenge intracellular NO similar to the levels as those in the normal group and downregulate the expression levels of CA9 mRNA (≈90%), thereby repolarizing the M1 macrophages into the M2 phenotype and increasing the expression levels of pro‐chondrogenic TGF‐*β*1 mRNA (≈1.3‐fold), and inhibiting chondrocyte apoptosis. Furthermore, in vivo experiments show that the NPs have great anti‐inflammation, cartilage protection and repair effects, thereby effectively alleviating OA progression in both monoiodoacetic acid‐induced early and late OA mouse models and a surgical destabilization of medial meniscus‐induced OA rat model. Therefore, the siCA9 and NO scavenger “two‐in‐one” delivery system is a potential and efficient strategy for progressive OA treatment.

## Graphical abstract

The carbonic anhydrase IX siRNA and nitric oxide (NO) scavenger in “two‐in‐one” nanocarriers is developed for progressive osteoarthritis (OA) therapy. A two‐pronged strategy is proposed for the regulation of intracellular/extracellular NO and hydrogen protons for reprogramming M1/M2 synovial macrophages, which holds promising application prospects for clinical translation of progressive OA treatment.

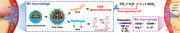

## 1 Introduction

Osteoarthritis (OA) is a chronic joint degenerative disease, one of the leading causes of disability worldwide, and has been listed as one of the “three killers” of human health by the World Health Organization (WHO). The prevalence of OA is increasing steadily, with 303 million adults being affected worldwide. OA has deleterious effects on the daily life of patients and is a great economic burden on public health. OA is characterized by synovial inflammation and articular cartilage destruction.^[^ ^1^ ^]^ Current pharmacological treatments (such as oral nonsteroidal anti‐inflammatory drugs, intra‐articular injected corticosteroids, and lubricants) merely relieve symptoms^[^ ^2^, ^3^ ^]^ but fail to hinder OA progression.^[^ ^4^ ^]^ Generally, synovial macrophages, key regulators of OA physiopathology,^[^ ^5^ ^]^ are maintained in an activated state in OA joints. Growing evidence has shown that dysfunctional synovial macrophages with an imbalance in the M1/M2 phenotype are significantly associated with synovial inflammation, cartilage destruction, and OA severity.^[^ ^6^ ^]^ Therefore, remodeling synovial macrophages is one of the most effective approaches to improve joint function and inhibit OA progression.

An increased M1/M2 phenotype ratio plays a critical role in the progression of OA. M2 macrophages secrete anti‐inflammatory cytokines and tissue repair factors that are beneficial for the resolution of inflammation and cartilage repair. In contrast, M1 macrophages secrete considerable amounts of proinflammatory cytokines (such as IL‐6, IL‐1*β*, and TNF‐*α*) and cartilage‐degrading enzymes (such as MMP and proteoglycanase) to aggravate synovial inflammation and cartilage destruction.^[^ ^7^ ^]^ Therefore, repolarizing M1 macrophages into the M2 phenotype is emerging as an effective OA treatment strategy based on different drugs, including NSAIDs (such as piroxicam, indomethacin, and celecoxib), corticosteroids, IL‐1 receptor antagonists, CO donors, and superoxide dismutases. Unfortunately, the repolarization efficiency of M1 macrophages by these therapeutic agents remains insufficient for regulating the severe inflammation in progressive OA.

Synovial inflammation is closely associated with the acidic microenvironment of OA. Normal synovial fluid and arthritic synovial fluid are characterized by pH ranges of 7.4–7.8 and 6.6–7.2, respectively.^[^ ^8^ ^]^ The inflammatory properties of M1 macrophages are influenced by changes in glucose metabolism in acidic and hypoxic environments. Thus, regulation of the acidic microenvironment may be a new therapeutic strategy for reprogramming the M1/M2 phenotype. Carbonic anhydrase IX (CA9) is a tumor marker responsible for the reversible hydration of carbon dioxide (CO_2_) to release protons (H^+^) and bicarbonate ions (HCO^3−^), regulating both intra‐ (pHi) and extracellular (pHe) pH balance and contributing to the hypoxic and acidic tumor microenvironment.^[^ ^9^ ^]^ CA9 inhibitors are widely used to suppress tumor growth and metastases,^[^ ^10^ ^]^ but their use in OA treatment has not been reported. In addition, various nitric oxide (NO) scavengers and iNOS inhibitors have exhibited strong anti‐inflammatory and macrophage reprogramming effects by suppressing intracellular and extracellular concentrations of NO because NO produced by M1 macrophages is 30‐fold higher than that produced by M2 macrophages.^[^ ^11^ ^]^ However, their use is limited to rheumatoid arthritis and early‐stage OA treatment. Therefore, we hypothesized that a two‐pronged scavenging strategy based on CA9 and NO might improve progressive OA by inhibiting intracellular and extracellular concentrations of NO and H^+^ to promote the reprogramming of synovial macrophages.

**Scheme 1:**
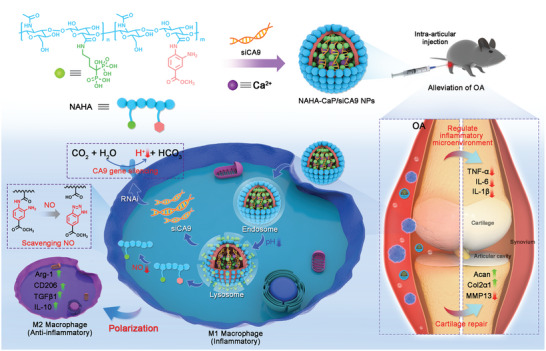
Preparation and mechanism of NAHA‐CaP/siCA9 NPs for treating OA. In the OA model mouse, the CA IX gene was downregulated and NO was scavenged in the synovial macrophages after intra‐articular injection of NAHA‐CaP/siCA9 NPs. Subsequently, these NPs reprogrammed proinflammatory M1 macrophages to the anti‐inflammatory M2 phenotype, and further inhibited the production of proinflammatory cytokines (TNF‐*α*, IL‐6, and IL‐1*β*). Furthermore, the levels of pro‐chondrogenic proteins (Acan and Col2*α*1) were increased and the levels of cartilage matrix degrading protein (MMP13) were decreased after the microenvironment was remodeled, thereby achieving protective and repaired action on cartilage.

In this study, we proposed a combination of CA9 siRNA (siCA9) and NO scavenger in a “two‐in‐one” nanocarrier (NAHA‐CaP/siCA9 NPs) for progressive OA therapy (**Scheme** **1**). siCA9 was encapsulated by calcium phosphate (CaP)/siRNA coprecipitation through coordination interactions between calcium ions and phosphate of siRNA. The NAHA polymer grafted with alendronate and *o*‐phenylenediamine on hyaluronic acid (HA) was used as the outer shell to stabilize the inner core of CaP/siRNA complexes. *O*‐phenylenediamine reacted with NO and decomposed into benzotriazole and a carboxyl group, resulting in consumption of NO. After intra‐articular injection, the NAHA‐CaP/siCA9 NPs were internalized into synovial M1 macrophages of OA joints, driving macrophage repolarization from the M1 to M2 phenotype by gene‐silencing effects of siCA9 and scavenging effects of *o*‐phenylenediamine in response to NO. We showed for the first time that CA9 was overexpressed in the synovial macrophages of OA joint tissues, which might serve as a novel therapeutic target for OA. These NPs effectively suppressed inflammation in the OA synovium and improved cartilage protection and repair, thereby attenuating OA progression. The in vivo therapeutic efficiency of NAHA‐CaP/siCA9 NPs was evaluated in monoiodoacetic acid (MIA)‐induced early‐stage OA mice, advanced OA mice, and destabilization of the medial meniscus (DMM) surgery‐induced OA rats. Our study demonstrates an innovative therapeutic strategy to effectively inhibit OA progression, with excellent potential for the clinical treatment of advanced OA.

## 2 Results and Discussion

### 2.1 Overexpression of CA9 in Synovial Macrophages of OA

**Figure 1:**
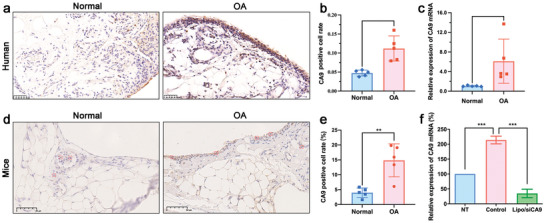
CA9 expression in OA synovial macrophages. a) Immunohistochemistry (IHC) images of synovium tissues in cartilage damaged or healthy knee joints from patients with OA (*n* = 5). Scale bar, 50 µm. b) Quantification of CA9‐positive cell rate in synovium tissues from patients with OA analyzed using IHC images (*n* = 5). c) The expression levels of CA9 mRNA in synovium tissues from patients with OA detected using qRT‐PCR assay (*n* = 5). d) IHC images of synovium tissues from healthy and MIA‐induced OA mouse models (*n* = 5). The red box referred to the representative synovial macrophages. Scale bar, 50 µm. e) Quantification of CA9‐positive cell rate in synovium tissues from healthy and OA mouse models analyzed using IHC images (*n* = 5). f) The expression levels of CA9 mRNA in the LPS‐activated RAW264.7 cells detected using qRT‐PCR assay (*n* = 3). “NT” referred to the normal RAW264.7 macrophages without LPS treatment, and the “Control” referred to the inflammatory RAW264.7 macrophages activated by LPS. Data are represented as mean ± SD. *p < 0.05, **p < 0.01, ***p < 0.001.

Immunohistochemistry (IHC) and quantitative real‐time PCR (qRT‐PCR) were performed on synovial tissues isolated from patients with OA undergoing total knee arthroplasty to evaluate the expression levels of CA9 protein in OA‐related synovial macrophages. As shown in **Figure** **1**a–c, compared with normal synovium samples, higher expression levels of CA9 protein (≈2‐fold) and mRNA (≈4.8‐fold) were observed in the synovium of patients with OA. We also investigated the expression of CA9 in synovial macrophages of mice with MIA‐induced OA using IHC. As shown in Figure 1d, higher CA9 expression levels (shown in brownish yellow) were observed in synovial macrophages (as indicated by the red box) from OA mouse models as compared to those in samples from healthy mice. The quantitative data of IHC further revealed ≈3.7‐fold higher CA9 expression levels in arthritic synovial macrophages than in healthy synovial macrophages (Figure 1e).

Furthermore, CA9 mRNA expression levels in murine RAW264.7 macrophages were also detected using qRT‐PCR. Synovial M1 macrophages are activated by stimulation with bacterial lipopolysaccharides (LPS) or cytokine interferon‐*γ* (IFN‐*γ*).^[^ ^12^ ^]^ As presented in Figure 1f, the activated macrophages (“control”) exhibited ≈2‐fold higher CA9 mRNA expression levels than the normal macrophages (“NT”). Interestingly, mRNA expression levels in the cells significantly downregulated (≈82%) by siCA9 transfected with the commercial reagent Lipofectamine 2000 after treatment for 24 h.

Taken together, the increase in CA9 levels in OA‐related synovium and LPS‐induced macrophages suggests that the CA9 protein might play a potential role in OA development, and the siCA9‐based delivery system could provide a novel strategy for effective OA therapy.

### 2.2 Preparation and Characterization of NAHA‐CaP/siRNA NPs

The assembly elements used for the NAHA‐CaP/siRNA NPs, such as HA, alendronate, and Ca^2+^, have great biosafety. HA, a natural joint lubricant synthesized in situ^[^ ^13^ ^]^ and oriented by CD44 targeting,^[^ ^14^ ^]^ shows great biocompatibility in inflamed joints, based on which its derivatives are often used as vehicles for drug delivery.^[^ ^15^ ^]^ In addition, unlike existing physically encapsulated NO scavengers, covalently linked *o*‐phenylenediamines were incorporated as part of the NAHA materials, facilitating their stable loading in the nanocarriers and durable retention at synovial sites.

The NAHA polymer grafted with alendronate and *o*‐phenylenediamine on HA was successfully synthesized, as described in Supporting Information (Figure S1, Supporting Information). The NAHA polymer was used as the outer shell to stabilize the inner core of the CaP/siRNA complexes. The AHA polymer grafted with alendronate on HA was synthesized as a control, which lacked NO‐scavenging capability. The structures of the corresponding materials were characterized using proton nuclear magnetic resonance spectroscopy (^1^H NMR) (Figure S2, Supporting Information).

**Figure 2:**
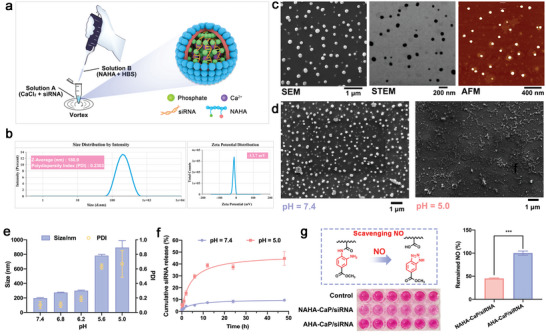
Characterization of NAHA‐CaP/siRNA NPs. a) Schematic illustration of the preparation method of nanoparticles. b) Size and zeta potential distribution of NPs detected using DLS. c) Representative SEM, STEM, and AFM images of NPs. d) SEM images of NPs under different pH conditions. Scale bars, 1 µm. e) The changes in particle size detected using DLS under different pH conditions (*n* = 3). f) The release curve of siRNAs from NPs under different pH conditions (*n* = 3). g) NO scavenging capability of NPs detected using the Griess assay (*n* = 6). Data are represented as mean ± SD. *p < 0.05, **p < 0.01, ***p < 0.001.

Both NAHA‐CaP/siRNA and AHA‐CaP/siRNA NPs were prepared using the nanoprecipitation method described previously.^[^ ^15^, ^16^ ^]^ Briefly, Solution B was established by dissolving NAHA or AHA materials in HBS buffer (including 50 × 10^−3^ m HEPES, 280 × 10^−3^ m NaCl, and 1.5 × 10^−3^ m Na_2_HPO_4_, pH = 7.4). Solution B was added dropwise to solution A (containing CaCl_2_ and siRNA) by vortexing (**Figure** **2**a). CaP/siRNA complexes were first obtained by the coordination interaction between calcium ions and phosphate ions, and then were further coated them with NAHA or AHA polymers to stabilize the rapidly growing calcium phosphate coprecipitation.

As shown in Figure 2b, Figure S3 (Supporting Information), and Table S1 (Supporting Information), both NAHA‐CaP/siRNA NPs and AHA‐CaP/siRNA NPs had uniform hydrodynamic sizes of ≈180 nm, a narrow size distribution (PDI ≈ 0.2), and zeta potentials of −13 mV, measured using dynamic light scattering (DLS). Transmission electron microscopy (TEM), scanning transmission electron microscopy (STEM), and atomic force microscopy (AFM) revealed that the NAHA‐CaP/siRNA NPs exhibited an average diameter of ≈50 nm with a uniform spherical shape and smooth surface morphology (Figure 2c). The average size shown in TEM, STEM, and AFM was significantly smaller than that of DLS, because the data of DLS was hydrodynamic size rather than the size of dehydrated particles measured in TEM, STEM, and AFM. In addition, the encapsulation efficiency of siRNA in the NAHA‐CaP/siCA9 NPs was around 96.8% based on a Ribogreen assay. Also, the loading efficiencies and protective effects of siRNAs in NPs were further investigated using a gel retardation assay after incubation of NAHA‐CaP/siRNA NPs with 50% fetal bovine serum (FBS) for different time points at 37 °C. As shown in Figure S4a (Supporting Information), the band of naked siRNAs almost disappeared in the electrophoresis images from 6 h, whereas a clear band of intact siRNAs was found in the NAHA‐CaP/siRNA group at 36 h. These results suggest the efficient encapsulation and great protective effects of siRNAs in NPs against enzymatic degradation in serum, indicating potential for feasible in vivo applications. The results of storage stability (Figure S4b, Supporting Information) showed no significant changes in the particle size (≈180 nm) of NAHA‐CaP/siRNA NPs at 4 °C within the 9‐d experimental period. The NAHA‐CaP/siRNA NPs exhibited a stable size distribution (<350 nm at 80 times) after dilution at different folds in HBS buffer (pH = 7.40) (Figure S4c, Supporting Information), indicating that these NPs had great anti‐dilution effects and would be beneficial for in vivo applications.

The intracellular disassembly of nanoparticles is essential for the release of siRNA to exert gene‐silencing effects. To investigate the pH‐responsive disassembly of NAHA‐CaP/siRNA NPs, changes in hydrodynamic size, morphology, and siRNA release profile under different pH conditions were measured. As shown in Figure 2e, the size of the NPs increased with a decrease in pH values. Especially, when the pH was dropped to 5.6 (similar to endosomes) and 5.0 (similar to lysosomes), the particle size (≈800 and ≈1000 nm, respectively) sharply increased, indicating that the NPs became loose as the acidity increased. Similarly, the SEM images also showed that the NPs maintained a spherical shape with an average diameter of 50 nm in pH = 7.4 media, whereas the NPs remarkably changed to an irregular shape accompanied by disassembly at pH = 5.0 (Figure 2d). Furthermore, the pH‐responsive disassembly of NPs promoted the release of siRNAs. At pH 7.4, the siRNAs barely leaked from the NPs (<10%); however, siRNAs were rapidly released at pH 5.0 (Figure 2f).

To investigate the NO‐scavenging ability of NAHA polymers and NAHA‐CaP/siRNA NPs, in vitro NO‐responsive changes after incubation of NAHA polymers or NPs with NO‐saturated solution were detected. The *o*‐phenylenediamine structure on NAHA reacts with NO and decomposes into benzotriazole and a carboxyl group, resulting in the consumption of NO (Figure 2g). After the addition of NO, the benzotriazole formed by the reaction was removed using dialysis, resulting in a 72.1% reduction in the integral proportion of *o*‐phenylenediamine (*δ* 7.7–8.0 ppm) in the ^1^H NMR spectrum (Figure S6, Supporting Information). Furthermore, to confirm the NO‐scavenging capability of NAHA‐CaP/siRNA NPs, the NO consumption after incubation of NPs with NO‐saturated solution was investigated using the Griess assay, which detects the nitrite ion (indicative of NO). As observed in Figure 2g, the NAHA‐CaP/siRNA NPs showed significantly reduced NO levels (≈55%) in NO solution, whereas AHA‐CaP/siRNA NPs had almost no change in NO consumption rate as compared with that of the control group. Correspondingly, the noticeably lighter color of the Griess reagent in the NAHA‐CaP/siRNA groups also confirmed the scavenging capability of these NPs. In addition, the particle size of NAHA‐CaP/siRNA NPs showed few changes in response to NO solubilized in distilled water, which might indicate that the NPs did not disassemble in the NO‐containing environment (Figure S7, Supporting Information).

These results revealed that the NAHA‐CaP/siRNA NPs possessed the appropriate size, negative charge, good siRNA encapsulation and stability, pH‐responsive disassembly, siRNA release, and NO‐scavenging effects, which are prerequisite for in vitro and in vivo applications.

### 2.3 Cellular Uptake and Lysosomal Escape of NPs

The cytotoxicity of siNC (negative control siRNA)‐loaded NPs in normal RAW264.7 macrophage cells was investigated before the in vitro experiments after incubation for 24 or 48 h. As shown in Figure S8 (Supporting Information), NAHA‐CaP/siNC and AHA‐CaP/siNC NPs had ≈100% cell viability, suggesting that these NPs had good biocompatibility in normal macrophages.

**Figure 3:**
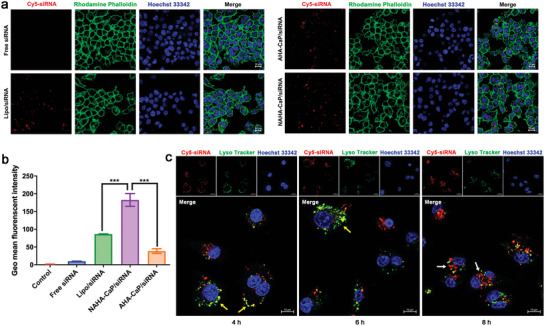
a) CLSM images of LPS‐activated RAW264.7 cells incubated with Cy5‐siRNA‐loaded NPs (red) at 37 °C for 6 h. The Rhodamine‐labeled phalloidin (green) was used to show cell skeleton and Hoechst 33342 (blue) for cell nucleus. Scale bars, 10 µm. b) Intracellular fluorescence intensities detected using flow cytometry after incubation of LPS‐activated RAW264.7 cells with the Cy5‐siRNA‐loaded NPs for 6 h (*n* = 3). c) Colocalization (yellow) of Cy5‐siRNA‐loaded NPs (red) and LysoTracker Red DND‐99‐stained endosome/lysosomes (green) in LPS‐activated RAW264.7 cells observed using CLSM images. Hoechst 33342 (blue) was used for staining the cell nucleus. Scale bars, 10 µm. The colocalization dots were indicated as yellow arrows, and the separated NPs were indicated as white arrows. Data are represented as mean ± SD. *p < 0.05, **p < 0.01, ***p < 0.001.

Efficient cellular uptake and rapid lysosomal escape of nanoparticles are essential for siRNA‐mediated gene silencing effects. As shown in confocal laser scanning microscopy (CLSM) images (**Figure** **3**a), NAHA‐CaP/siRNA NPs showed higher intracellular red fluorescence (Cy5‐labeled siRNAs) in LPS‐activated RAW264.7 cells after 6 h of incubation when compared with the free siRNA group, and even higher levels than that of AHA‐CaP/siRNA and Lipo/siRNA groups. Consistently, flow cytometry results (Figure 3b) also indicated that the fluorescence intensity in the cells treated with NAHA‐CaP/siRNA NPs was 2.1 and 4.8 times higher than that of Lipo/siRNA and AHA‐CaP/siRNA, respectively. These results suggested that the improved cellular uptake efficiency of NAHA‐CaP/siRNA NPs was probably attributed to the rigidity of these NPs based on the modification of *o*‐phenylenediamine. AHA‐CaP/siRNA NPs exhibited relatively low cell internalization due to their lack of hydrophobic *o*‐phenylenediamine modification. As reported, rigidity of nanoparticles could dramatically alter the cellular uptake efficiency, with more rigid nanoparticles able to move more easily through membranes.^[^ ^17^ ^]^

To further investigate whether NAHA‐CaP/siRNA NPs could escape from endosomes/lysosomes following cell internalization, intracellular fluorescence distribution was measured using CLSM images. As seen in Figure 3c, most of the colocalized (yellow) Cy5‐labeled siRNA (red) and LysoTracker‐labeled lysosomes (green) were observed in the cytoplasm after incubation of cells with NPs for 4 and 6 h. A large number of red fluorescent dots (as indicated by the white arrows) distinct from the green fluorescence dots after 8 h of incubation, indicating that these NPs could achieve lysosomal escape into the cytoplasm at 8 h.

### 2.4 In Vitro Anti‐Inflammatory Effects of NAHA‐CaP/siCA9 NPs by NO Scavenging and Gene Silencing

**Figure 4:**
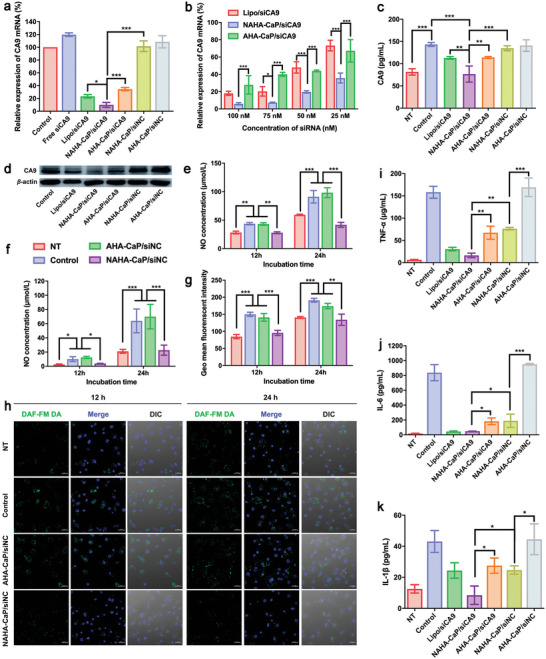
a) Relative expression levels of CA9 mRNA detected using qRT‐PCR after incubating LPS‐activated RAW264.7 cells with different formulations for 24 h (siRNA concentration, 100 × 10^−9^ m) (*n* = 3). b) Relative expression levels of CA9 mRNA detected using qRT‐PCR after incubating LPS‐activated RAW264.7 cells with different formulations for 24 h. The final concentration of siRNA was 25, 50, 75, and 100 × 10^−9^ m, respectively (*n* = 3). c) The expression levels of CA9 protein detected using ELISA (*n* = 3) and d) Western blotting assay after incubation of cells with different formulations (siRNA concentration, 100 × 10^−9^ m). e) Extracellular and f) intracellular NO‐scavenging ability of NAHA‐CaP/siRNA NPs in LPS‐activated RAW264.7 cells detected using Griess assay (*n* = 3). g) Intracellular NO‐scavenging ability of NAHA‐CaP/siRNA NPs in LPS‐activated RAW264.7 cells detected using flow cytometer (*n* = 3) and h) CLSM. Nuclei and NO were stained with Hoechst33342 (blue) and DAF‐FM DA (green), respectively. (Scale bar = 20 µm). The concentrations of i) TNF‐*α*, j) IL‐6, and k) IL‐1*β* after incubation of cells with different formulations for 24 h (*n* = 3). “NT” referred to the normal RAW264.7 macrophages without LPS treatment. Data are represented as mean ± SD. *p < 0.05, **p < 0.01, ***p < 0.001.

To investigate the gene‐silencing effects of NAHA‐CaP/siRNA NPs, CA9 mRNA levels were detected using qRT‐PCR in LPS‐activated RAW264.7 cells. As shown in **Figure** **4**a, the NAHA‐CaP/siCA9 group had significantly higher gene silencing effects (≈90%) than Lipo/siCA9 (≈77%) and AHA‐CaP/siCA9 (≈65%). In contrast, the scrambled siRNA‐loaded NAHA‐CaP/siNC group did not show any downregulation of CA9 mRNA. These results demonstrated that NAHA‐CaP/siCA9 NPs effectively silenced CA9 mRNA in LPS‐activated RAW264.7 cells. In addition, dose‐dependent gene silencing was observed in the siCA9‐loaded groups (Figure 4b). The NAHA‐CaP/siCA9 group exhibited the strongest downregulation effects at each siRNA concentration (100 × 10^−9^ m, ≈94%; 75 × 10^−9^ m, ≈93%; 50 × 10^−9^ m, ≈80%; 25 × 10^−9^ m, ≈64%).

To determine the effects of downregulation of CA9 proteins by NPs, ELISA and western blot assays were performed. Compared with the NT group (the cells without LPS treatment), the LPS‐activated RAW264.7 cells showed a 76% increase in CA9 protein expression level (Figure 4c), which was consistent with the CA9 mRNA levels as shown in Figure 1d. NAHA‐CaP/siCA9 NPs showed a stronger downregulation of CA9 protein (46.4%) than Lipo/siCA9 (21.1%) and AHA‐CaP/siCA9 NPs (20.3%), which was in accordance with the qRT‐PCR results. The significant downregulation of CA9 protein by NAHA‐CaP/siCA9 NPs was also validated using western blotting (Figure 4d). These results demonstrated that NAHA‐CaP/siCA9 NPs could efficiently inhibit CA9 protein in LPS‐activated RAW264.7 cells.

To investigate the NO‐scavenging ability of NPs in vitro, the concentrations of extracellular and intracellular NO were detected using Griess assay, flow cytometry, and CLSM after treating cells with NAHA‐CaP/siNC NPs. As shown in Figure 4e,f, the concentrations of both intracellular and extracellular NO in the LPS‐activated cells (control group) were ≈1.5 fold higher than those in the NT group, as detected using the Griess assay after 12 h of incubation. As the incubation time increased to 24 h, the concentrations of both intracellular and extracellular NO in the control group were further increased, which were ≈3‐fold and 4‐fold higher than those in the NT group. These results indicated that the RAW264.7 cells activated by LPS could induce high levels of NO production. Notably, NAHA‐CaP/siNC groups significantly reduced the concentrations of extracellular and intracellular NO to the same levels as those observed in the NT groups at 12 and 24 h of incubation. In contrast, NO levels in the AHA‐CaP/siNC group were not significantly different from those in the control group. These results demonstrate that NAHA‐CaP/siRNA NPs consume intracellular and extracellular NO, which is attributed to the reaction between NO and *o*‐phenylenediamine. The concentrations of intracellular NO were also detected using flow cytometry after the cells were treated with the NO fluorescent probe 3‐amino, 4‐aminomethyl‐2′, 7′‐difluorescein, diacetate (DAF‐FM DA). The AHA‐CaP/siNC NPs did not show obvious NO‐scavenging efficacy after 12 or 24 h of incubation, while the NAHA‐CaP/siNC groups decreased the concentration of NO to levels similar to those observed in the NT groups at 12 or 24 h of incubation (Figure 4g). Consistently, fewer intracellular NO fluorescent signal dots (green) were observed in the NAHA‐CaP/siNC groups than in the AHA‐CaP/siNC and control groups, as shown in the CLSM images, indicating the excellent intracellular NO‐scavenging effects of NAHA‐CaP/siNC NPs (Figure 4h).

High levels of NO can increase the production of proinflammatory cytokines and are closely related to synovial inflammation in OA.^[^ ^18^ ^]^ To investigate the anti‐inflammatory effects of NAHA‐CaP/siRNA NPs, the in vitro concentrations of major proinflammatory cytokines were measured using ELISA. As shown in Figure 4i–k, the NAHA‐CaP/siNC group showed better inhibitory effects on three inflammatory cytokines (inhibition rate of TNF‐*α*: ≈52%; IL‐1*β*: ≈43%; IL‐6: ≈77%) than the AHA‐CaP/siNC and control groups, demonstrating that the NAHA‐CaP/siNC group could reduce the secretion of proinflammatory factors by NO‐scavenging effects. In addition, compared with the control group, the siCA9‐loaded groups significantly reduced the levels of three cytokines, indicating that the silencing of CA9 protein by siRNA could inhibit the secretion of these inflammatory factors. Moreover, NAHA‐CaP/siCA9 NPs exhibited the strongest inhibitory effects on TNF‐*α* (≈90%), IL‐1*β* (≈80%), and IL‐6 (≈94%). These results revealed that the anti‐inflammatory efficacy of NAHA‐CaP/siCA9 NPs resulted from a synergistic mechanism of NO‐scavenging and CA9‐silencing.

In addition to inhibiting the production of proinflammatory factors, the potent anti‐inflammatory effects of NAHA‐CaP/siCA9 NPs were associated with the inhibition of proinflammatory signaling pathways. Several studies have indicated that the nuclear factor kappa B (NF‐*κ*B),^[^ ^19^ ^]^ toll‐like receptor (TLR),^[^ ^20^ ^]^ and mitogen‐activated protein kinase (MAPK) pathways^[^ ^21^ ^]^ are activated during inflammation.^[^ ^22^ ^]^ To investigate the inhibitory effects of NAHA‐CaP/siCA9 NPs on these pathways, the expression levels of p38 MAPK, NF‐*κ*B (p50/p65), and MyD88 signaling were evaluated using western blotting and ELISA in LPS‐activated macrophage cells. As shown in Figures S9 and S10 (Supporting Information), compared to the control group, the NAHA‐CaP/siNC group effectively inhibited the expression of NF‐*κ*B p50/p65, phosphorylated p65, phosphorylated p38, and MyD88, which was associated with its NO‐scavenging ability. Moreover, the NAHA‐CaP/siCA9 NPs had better inhibitory effects on these pathways than the NAHA‐CaP/siNC and AHA‐CaP/siCA9 groups, suggesting that the synergistic anti‐inflammatory effects were attributed to the functions of NO‐scavenging and CA9 gene silencing in NPs.

### 2.5 In Vitro M1 to M2 Macrophage Repolarization Induced by NAHA‐CaP/siCA9 NPs

As previously reported, proinflammatory cytokines are primarily produced by M1 macrophages.^[^ ^7a^ ^]^ M2 macrophages produce anti‐inflammatory factors and respond to tissue repair.^[^ ^23^ ^]^ Macrophage M1/M2 polarization dynamically adapts to changes in the microenvironment.^[^ ^24^ ^]^ Therefore, switching macrophages from M1 to M2 phenotype is a potential strategy for the treatment of OA.

**Figure 5:**
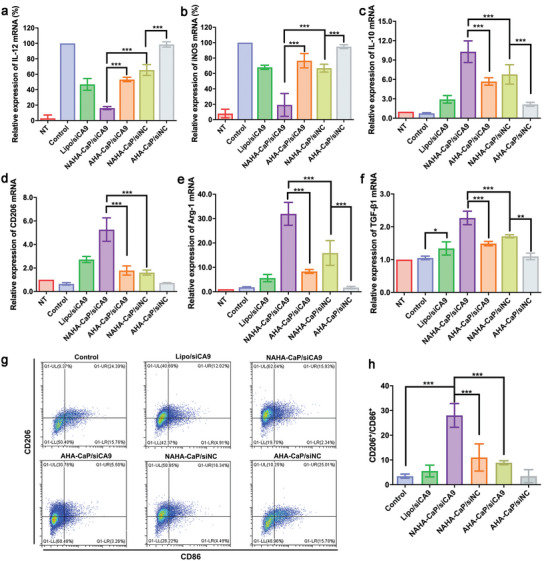
Relative expression levels of M1 macrophage markers a) IL‐12 and b) iNOS mRNA quantified using qRT‐PCR. Data was shown as relative expression to the control group (*n* = 3). Relative expression levels of M2 macrophage markers c) IL‐10, d) CD206, and e) Arg‐1 mRNA quantified using qRT‐PCR. Data was shown as normalized fold expression relative to the NT group (*n* = 3). f) Relative expression levels of pro‐chondrogenic gene TGF‐*β*1 mRNA detected using qRT‐PCR after incubating LPS‐activated RAW264.7 cells with different formulations for 24 h (*n* = 3). g) Representative images of macrophage subtypes by staining CD86 (M1 macrophage marker) and CD206 (M2 macrophage marker) evaluated using flow cytometry. h) Ratio of the numbers of CD206‐positive to CD86‐positive macrophage cells measured using flow cytometry (*n* = 3). “NT” referred to the normal RAW264.7 macrophages without LPS treatment. Data were shown as mean ± SD. *p < 0.05, **p < 0.01, ***p < 0.001.

qRT‐PCR, western blotting, and ELISA were used to examine whether NAHA‐CaP/siCA9 NPs could repolarize LPS‐activated macrophages from the M1 phenotype to the M2 phenotype. IL‐12 and iNOS are representative markers of the M1 phenotype. Arg‐1, IL‐10, and CD206 are regarded as representative markers of the M2 phenotype. As shown in **Figure** **5**a,b, the expression levels of IL‐12 and iNOS mRNA increased after the cells were stimulated by LPS. For M1 phenotype detection, the relative expression levels in the control group were set to 100%. NAHA‐CaP/siCA9 NPs exhibited stronger downregulatory effects on the mRNA expression of IL‐12 (≈84%) and iNOS (≈81%) than AHA‐CaP/siCA9 NPs (*p* < 0.001) and NAHA‐CaP/siNC NPs (*p* < 0.001). For M2 phenotype detection, the relative expression levels in the NT group were set to 100%. Compared with the control group, NAHA‐CaP/siCA9 NPs upregulated the expression of IL‐10, Arg‐1, and CD206 mRNA by 12.6‐, 16.2‐, and 7.1‐fold, respectively, which were higher than those of other formulations (Figure 5c–e). The expression levels of intracellular IL‐10 protein were detected using ELISA. As shown in Figure S11 (Supporting Information), NAHA‐CaP/siCA9 NPs showed higher IL‐10 expression levels than the other groups, which was consistent with the qRT‐PCR results. The results of mRNA and protein expression demonstrated that M1 phenotype macrophages were repolarized to the M2 phenotype by NAHA‐CaP/siCA9 NPs.

To verify the capability of NAHA‐CaP/siCA9 NPs to reprogram macrophages, the subtypes of RAW264.7 cells were analyzed using flow cytometry after staining for CD86 (M1 marker) and CD206 (M2 marker). A significant decrease in CD86‐positive cell subtypes and increase in CD206‐positive cell subtypes was observed in M1 macrophages after AHA‐CaP/siCA9 or NAHA‐CaP/siNC treatment (Figure 5g). Specifically, after incubation with NAHA‐CaP/siCA9 NPs, the number of CD86‐positive cells decreased from 15.76% to 2.34%, and the number of CD206‐positive cells increased from 9.37% to 62.04%. The ratio of CD206^+^/CD86^+^ cells in the NAHA‐CaP/siCA9 group (≈28.0) was significantly higher than that in the NAHA‐CaP/siNC (≈11.0) and AHA‐CaP/siCA9 groups (≈8.9) (Figure 5h), demonstrating that the NAHA‐CaP/siCA9 NPs promoted macrophage repolarization from the M1 to M2 phenotype by synergistic CA9 silencing and NO scavenging effects. STAT6 is an important transcription factor involved in M2 polarization.^[^ ^25^ ^]^ Therefore, the phosphorylation levels of STAT6 (p‐STAT6) were evaluated using western blotting after the treatment of cells with NPs. The NAHA‐CaP/siCA9 NPs promoted the expression of p‐STAT6 in LPS‐activated macrophages (Figure S12, Supporting Information), indicating that the NAHA‐CaP/siCA9 NP‐induced phenotype alteration of macrophages from M1 to M2 was closely related to the activation of STAT6 pathways.

Taken together, these results demonstrate that the NO scavenger and siCA9 codelivering NAHA‐CaP/siCA9 NPs effectively inhibited M1 activation and repolarized M1 macrophages to the M2 phenotype, thereby regulating inflammatory cytokine expression to exert anti‐inflammatory activity.

### 2.6 In Vitro Cartilage Protection and Repair Effects of NAHA‐CaP/siCA9 NPs

It is important to not only control articular inflammation but also improve the local microenvironment to support chondrogenesis and prevent chondrocytes from OA‐induced apoptosis^[^ ^26^ ^]^ to effectively repair damaged articular cartilage caused by OA. The results demonstrated that NAHA‐CaP/siCA9 NPs exhibited an inhibitory effect on inflammation mediated by M1 macrophages and promoted macrophage polarization towards the M2 phenotype, which might alter the inflammatory microenvironment to support chondrogenesis. Interestingly, the expression levels of TGF‐*β*1 mRNA in LPS‐induced RAW264.7 cells, treated with NAHA‐CaP/siCA9 NPs, were the highest among all the test groups (Figure 5f). Considering that TGF‐*β*1 could be beneficial for repairing damaged articular cartilage as one of the important pro‐chondrogenic cytokines,^[^ ^27^ ^]^ NAHA‐CaP/siCA9 NPs might be beneficial for establishing a pro‐chondrogenic microenvironment based on the pharmacological effects of TGF‐*β*1.

**Figure 6:**
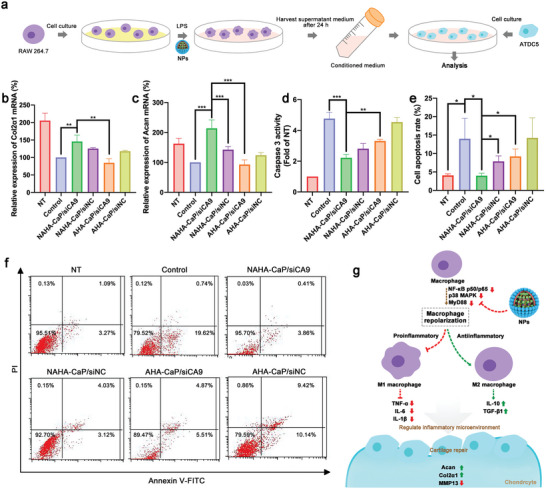
a) Schematic illustration for the conditioned culture of ATDC5 cells. Relative mRNA expression levels of b) Col2*α*1 and c) Acan after incubation of ATDC5 cells with NP‐treated macrophage conditioned medium for 48 h (*n* = 3). Data were shown as normalized fold expression relative to the control group. d) Caspase 3 activities after incubation of ATDC5 cells with NP‐treated macrophage conditioned medium (*n* = 3). Data were shown as normalized fold expression relative to the NT group. e) Representative images of ATDC5 cell apoptosis and f) apoptosis rate after incubation in conditioned medium of macrophages treated with different NPs detected using flow cytometry (*n* = 3). g) Schematic illustration of the in vitro mechanisms of NAHA‐CaP/siCA9 NPs. Data are represented as mean ± SD. *p < 0.05, **p < 0.01, ***p < 0.001.

The activation of TGF‐*β*/Smad signaling pathways by elevated TGF‐*β*1 in M2 macrophages could regulate the expression of Col2*α*1 and Acan, thus promoting cartilage repair.^[^ ^28^ ^]^ To evaluate the pro‐chondrogenic ability of TGF‐*β*1 induced by NAHA‐CaP/siCA9 in vitro, the expression levels of type II collagen (Col2*α*1) and proteoglycan (Acan), the representative components in the cartilage matrix associated with cartilage repair, were measured in chondrogenic ATDC5 cells using qRT‐PCR. Conditioned medium (CM) from NP‐treated LPS‐activated macrophages was collected for ATDC5 cell culture (**Figure** **6**a). As shown in Figure 6b,c, lower expression levels of Col2*α*1 and Acan mRNA were observed in ATDC5 cells cultured with CM from LPS‐induced macrophages (control group) than in the NT group. This might be attributed to damage to the prochondrogenic microenvironment induced by inflammatory cytokines or mediators produced by LPS‐activated M1 macrophages. After ATDC5 cells were cultured in CM from NAHA‐CaP/siCA9 NP‐treated macrophages, the mRNA expression levels of Col2*α*1 and Acan markedly increased by 45.8% and 121.9%, respectively, which were significantly higher than those in the NAHA‐CaP/siNC and AHA‐CaP/siCA9 groups. These results may be associated with the increased expression of TGF‐*β*1 in NAHA‐CaP/siCA9 NP‐treated macrophages.

Caspase 3 functions as a key effector in apoptotic cell death, and its inhibition can effectively prevent the initiation of cell apoptosis pathways.^[^ ^29^ ^]^ To further evaluate the anti‐apoptotic effects of TGF‐*β*1 induced by NAHA‐CaP/siCA9 NPs, the Caspase 3 activity and cell apoptosis rate of ADTC5 cells were measured after incubation with NPs‐treated macrophages CM. As shown in Figure 6d, compared with the NT group, the control group showed 3.8‐fold increase in Caspase 3 activity. Interestingly, the NAHA‐CaP/siCA9 group showed the highest inhibition rate (53.40%) of Caspase 3 activity among all NP‐treated groups. As shown in Figure 6e,f, the NAHA‐CaP/siCA9 group showed a significantly lower cell apoptosis rate than the other NP‐treated groups, even remaining comparable to that of the NT group. TGF‐*β*1 inhibits apoptosis, and the Fas/FasL pathway might be involved in this process. Considering that Caspase 3 could be triggered by the Fas/FasL pathways, it was speculated that the protective effects of TGF‐*β*1 in the NAHA‐CaP/siCA9 group against inflammatory macrophage‐induced cartilage destruction were associated with the TGF‐*β*1‐Fas/FasL‐Caspase 3 pathway. These results demonstrated that NAHA‐CaP/siCA9 NPs could prevent chondrocytes from inflammatory macrophage‐induced apoptosis, which might have protective effects against cartilage damage in OA in vivo. Additionally, the cytotoxicity of siRNA‐loaded different NPs in primary chondrocytes of mice was also investigated using CCK‐8 assay after incubation for 24 or 48 h. As shown in Figure S18 (Supporting Information), all NPs had ≈100% cell viability, suggesting that these NPs had good biocompatibility and biosafety in primary chondrocytes.

The increased expression of TGF‐*β*1 in M2 macrophages induced by NAHA‐CaP/siCA9 NPs enhanced cartilage matrix component expression to support cartilage repair and suppressed apoptosis of chondrocytes to provide cartilage protection (Figure 6g). These NPs may have great potential in mitigating OA progression in vivo.

### 2.7 The Inhibition Effects of NPs on Cartilage Damage and Synovitis in Early‐Stage OA Mice

OA is characterized by synovial inflammation, osteophyte formation, progressive degradation of the articular cartilage, and subchondral bone remodeling.^[^ ^30^ ^]^ The disease can undergo progressive evolution over time, leading to worsening severity and symptoms. At the early stage of OA, synovial inflammation acts as a key factor during OA pathogenesis owing to the activation and polarization of M1 macrophages induced by danger‐associated molecular patterns (DAMPs), which can produce proinflammatory cytokines.^[^ ^21^, ^31^ ^]^ Subsequently, polarized M1 macrophages alter intercellular signaling pathways, including TGF‐*β* and NF‐*κ*B signaling, which promote the degradation of extracellular matrix (ECM) components and further exacerbate cartilage degeneration.^[^ ^6a^ ^]^ These changes, in turn, result in repeated cycles of inflammation and cartilage degradation, which drive into the later stages of OA.^[^ ^30^ ^]^ To evaluate the therapeutic effects of NAHA‐CaP/siCA9 NPs on synovial inflammation and cartilage damage in vivo, three OA models with different stages of severity were established.

**Figure 7:**
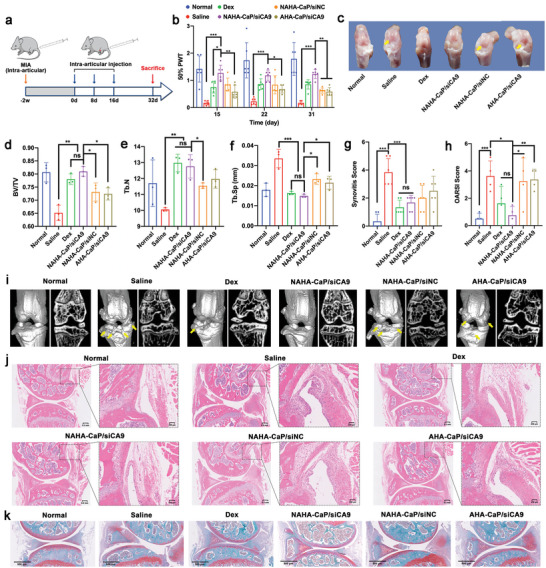
a) Schematic illustration of the experimental design in C57BL/6 mice with early‐stage OA. b) The paw withdrawal threshold evaluated using von Frey assay during the treatment (*n* = 6). c) Representative images of the macroscopic observation of the knee joints isolated from mice. The yellow arrows point to scratches or breakages. d) The quantitative measurements in bone volume fraction (BV/TV), e) trabecular bone number (Tb.N), and f) trabecular separation (Tb.Sp) detected using microCT (*n* = 3). g) Synovitis score of H&E‐stained sections (*n* = 6). h) Cartilage OARSI score in Safranin O‐fast green‐stained sections (*n* = 4). i) The representative microCT bone remodeling 3D and microCT 2D images of the mice knee joints. j) H&E staining images of mice knee joint tissues with early‐stage OA. Scale bars were 0.2 mm and 250 µm, respectively. k) Safranin O‐fast green staining images of mice knee joint tissues with early‐stage OA. Scale bars, 500 µm. Data are represented as mean ± SD. *p < 0.05, **p < 0.01, ***p < 0.001.

Two weeks following a single intra‐articular injection of 1% (w/v) MIA, an early‐stage OA mouse model was established, and the mice were randomly divided into five groups and treated by intra‐articular injection of different formulations at a siRNA dose of 0.5 mg kg^−1^. Healthy mice were used as controls. Dexamethasone (Dex), a representative anti‐inflammatory drug, was used as a positive control which is widely used to alleviate OA clinically to eliminate inflammation and relieve pain.^[^ ^32^ ^]^ All mice were injected every eight days until they were sacrificed on day 32 (**Figure** **7**a). During the experimental period, mechanical allodynia in mice was assessed by applying von Frey hairs to the plantar surface of the hind paw and analyzed using mechanical withdrawal thresholds. As shown in Figure 7b, compared with the normal group, the saline group showed significantly decreased paw withdrawal threshold (PWT), which suggested mechanical allodynia caused by OA pain. Whereas, all formulation‐treated groups greatly increased the PWT of mice, especially the NAHA‐CaP/siCA9 group, which exhibited the highest level of PWT throughout the experimental period, implying that the NAHA‐CaP/siCA9 NPs treatment could effectively relieve OA pain.

Macroscopic observations, computed tomography (microCT), hematoxylin and eosin (H&E), and Safranin O‐fast green staining assays were performed to evaluate the therapeutic effects of NAHA‐CaP/siCA9 NPs on synovial inflammation and cartilage damage at the end of the treatment. As shown in Figure 7c, cartilage loss and a rough articular surface were observed in the saline group; however, in the NAHA‐CaP/siCA9 group, the articular surface was relatively smooth with little wear, which suggested the cartilage protective effects of NPs. To further investigate subchondral bone changes, including thickening of the subchondral bone plate, osteophyte formation, and subchondral trabecular bone sclerosis, microCT scanning with both 2D images and 3D reconstruction was performed. Changes in bone volume/total volume (BV/TV), trabecular separation (Tb.Sp), and trabecular bone number (Tb.N) were also quantified to assess the degradation of articular cartilage and subchondral bone. As shown in Figure 7i, significant articular cartilage destruction and massive osteophyte formation with an unsmooth bone surface were observed in the saline group, while effectively suppressing cartilage defects and almost no osteophyte formation with a smooth bone surface was observed after treatment with NAHA‐CaP/siCA9 NPs. The number and volume of osteophytes in the NAHA‐CaP/siCA9 group were significantly lower than those in the NAHA‐CaP/siNC, AHA‐CaP/siCA9, and Dex groups. Moreover, a higher BV/TV, an indicator of changes in bone mass, was observed in the NAHA‐CaP/siCA9 group than in the NAHA‐CaP/siNC and AHA‐CaP/siCA9 groups (Figure 7d). In addition, among the NP‐treated groups, significant changes in trabecular parameters (highest Tb.N and lowest Tb.Sp) were observed in the NAHA‐CaP/siCA9 group (Figure 7e,f). It is noteworthy that the NAHA‐CaP/siCA9 group was not significantly different from the normal and Dex groups in terms of BV/TV, Tb.N, and Tb.Sp. These results indicated that NAHA‐CaP/siCA9 NPs could effectively prevent cartilage degradation and destruction to maintain bone mass and trabecular structure in a mouse model of early‐stage OA, suggesting that the excellent therapeutic efficacy came from the synergistic effects of the NO scavenger and siCA9.

H&E‐stained sections showed severe synovial hyperplasia, increased thickness of synovial lining cell layers, large numbers of inflammatory cells, and bone and cartilage destruction in the saline group (Figure 7j). The NAHA‐CaP/siCA9 group showed markedly reduced symptoms compared with the saline group. A lower synovitis score was observed in the NAHA‐CaP/siCA9 group, which was similar to that in the Dex group (Figure 7g). Safranin O‐fast green staining of isolated knee joints was performed to investigate the degradation of articular cartilage and the loss of ECM. As shown in Figure 7k, the surface of the normal cartilage was ordered and smooth, and proteoglycans (safranin O staining) were evenly distributed in the articular cartilage ECM. In contrast, typical features of OA were observed in the saline group, such as thinning of cartilage, loss of staining, and the apparent destruction of normal structures in extensive regions. Compared with the saline group, the NAHA‐CaP/siCA9, NAHA‐CaP/siNC, and AHA‐CaP/siCA9 groups showed improvements in morphological changes, matrix staining, and cartilage integrity. The NAHA‐CaP/siCA9 group had larger positive areas in safranin O staining with smooth and intact cartilage surfaces, which were closer to that of the normal group. Based on histological analysis, the NAHA‐CaP/siCA9 group recorded the lowest Osteoarthritis Research Society International (OARSI) score (Figure 7h), demonstrating the notable therapeutic effect of NAHA‐CaP/siCA9 NPs in reducing cartilage destruction in mice with early‐stage OA.

**Figure 8:**
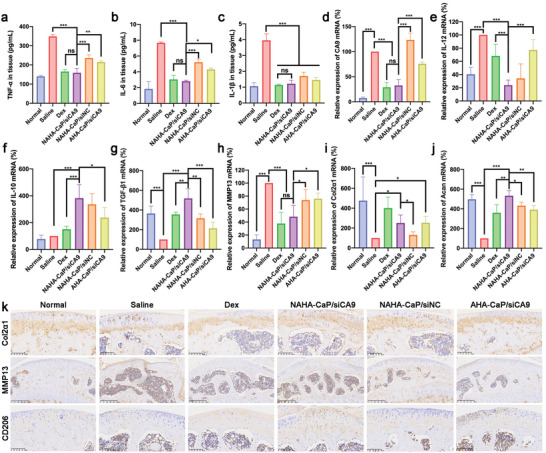
The concentrations of inflammatory factors a) TNF‐*α*, b) IL‐6, and c) IL‐1*β* in knee joint tissues isolated from mice with early‐stage OA (*n* = 3). Relative expression levels of d) CA9, e) IL‐12, f) IL‐10, g) TGF‐*β*1, h) MMP13, i) Col2*α*1, and j) Acan mRNA in mice knee joint tissues. Data were shown as normalized expression relative to the saline group (*n* = 5). k) The expression of Col2*α*1, MMP13 and CD206 proteins in the joint tissues detected by immunohistochemistry. Data are represented as mean ± SD. *p < 0.05, **p < 0.01, ***p < 0.001.

To investigate the anti‐inflammatory activity of NPs in vivo, the levels of proinflammatory cytokines in OA joint tissues and serum from mice were measured using ELISA. As shown in **Figure** **8**a–c, the levels of TNF‐*α*, IL‐6, and IL‐1*β* in the NAHA‐CaP/siCA9‐treated OA joints displayed a remarkable reduction as compared with those of other groups and even showed no significant difference from those of the normal group. Meanwhile, the levels of these proinflammatory cytokines in the serum were also dramatically decreased in the NAHA‐CaP/siCA9 group compared with the saline group (Figure S13, Supporting Information). Notably, the inflammation‐inhibitory effects of NAHA‐CaP/siCA9 NPs both in OA joints and serum were similar to those of Dex. These results revealed that NAHA‐CaP/siCA9 NPs could efficiently alleviate OA inflammation by scavenging NO and downregulating CA9 expression, providing evidence for the protective effects of NAHA‐CaP/siCA9 NPs against inflammatory mediator‐mediated cartilage destruction.

To further elucidate the in vivo anti‐inflammatory and cartilage repair mechanisms of NPs, ex vivo joint tissues of mice were collected for qRT‐PCR and immunohistochemistry assays. For the PCR results, the levels in the saline group were regarded as 100%. As shown in Figure 8d, the expression level of CA9 mRNA in the saline group was significantly higher than that in the normal group, confirming that elevated expression of CA9 was induced in OA joints. NAHA‐CaP/siCA9 NPs significantly inhibited CA9 mRNA levels by 67.5%, which was significantly better than that of AHA‐CaP/siCA9 NPs (24.4%). Surprisingly, the Dex group also showed an inhibitory effect on CA9 mRNA expression, which may be related to the indirect inhibitory effect on HIF‐1*α*.^[^ ^33^ ^]^ The expression levels of M1 macrophage marker (IL‐12) and M2 macrophage marker (IL‐10) mRNA are shown in Figure 8e,f, respectively. Compared with the saline group, the NAHA‐CaP/siCA9 group showed reduced IL‐12 mRNA levels by ≈76% and increased IL‐10 mRNA levels by ≈2.8‐folds, which was significantly higher than those in the other groups. In addition, immunohistochemistry results also showed that NAHA‐CaP/siCA9 NPs upregulated the number of CD206 positive cells (M2‐type macrophages) in the synovium (Figure 8k). These data confirmed the stimulatory effects of NAHA‐CaP/siCA9 NPs on the polarization of macrophages to the M2 phenotype. The NAHA‐CaP/siCA9 group exhibited the highest expression level of pro‐chondrogenic (TGF‐*β*1) mRNA among the various treatment groups (Figure 8g), which was beneficial for chondrogenesis. These results were consistent with the in vitro qRT‐PCR results. It is noteworthy that the NAHACaP/siCA9 group had stronger downregulation of IL‐12 mRNA (*p* < 0.001)) and upregulation of IL‐10 (*p* < 0.001) and TGF‐*β*1 mRNA (*p* < 0.05) than the Dex group, although both groups had similar anti‐inflammatory efficacy. These results suggest that NAHA‐CaP/siCA9 NPs have more potential to repolarize M1 macrophages into the M2 phenotype and promote chondrogenesis. Furthermore, proteoglycan and type II collagen are important cartilage matrix components that are responsible for the integrity of cartilage tissue.^[^ ^34^ ^]^ Therefore, the increased levels of these cytokines are necessary for the amelioration of cartilage degeneration. MMP13 is a matrix metalloproteinase expressed by hypertrophic chondrocytes that degrades the cartilage matrix.^[^ ^35^ ^]^ The expression levels of these cartilage matrix‐related mRNA and proteins in the joint tissues were detected using qRT‐PCR and immunohistochemistry, respectively. The saline group showed increased expression levels of MMP13 mRNA and decreased levels of Col2*α*1 and Acan mRNA when compared with the normal group, implying the degradation of the cartilage matrix (Figure 8h–j). The NAHA‐CaP/siCA9 NPs showed significantly lower MMP13 mRNA expression levels and higher Col2*α*1 and Acan mRNA expression levels than the NAHA‐CaP/siNC and AHA‐CaP/siCA9 groups. Specifically, the NAHA‐CaP/siCA9 group showed stronger Acan mRNA expression than the Dex group, which indicated that NAHA‐CaP/siCA9 NPs may have better cartilage repair effects. In addition, immunohistochemical staining showed positive expression of MMP13 (dark brown) on the cartilage surface in the saline group (Figure 8k). The NAHA‐CaP/siCA9 group showed a significant decrease in MMP13 expression compared with the other groups. The expression levels of Col2*α*1 protein in cartilage tissues from the NAHA‐CaP/siCA9 group were also significantly higher than those in the other groups. These results suggest that NAHA‐CaP/siCA9 NPs could reduce the production of proinflammatory cytokines by M1 macrophages, promote macrophage repolarization towards the M2 phenotype, and induce cartilage matrix component expression, thereby effectively protecting cartilage from damage and promoting cartilage repair in OA treatment.

The conventional strategy for treating OA is using corticosteroids such as Dex, which can alleviate the inflammatory response and reduce the degradation of the chondrocyte extracellular matrix. These in vivo results also confirmed the anti‐inflammatory and cartilage‐protective effects of Dex in early stage of OA. However, steroids, including Dex, do not target the underlying cause of the disease and are not recommended for long‐term management because they increase the risk of requiring joint replacement and are associated with chondrotoxicity.^[^ ^36^ ^]^ Notably, compared with the Dex group, the NAHA‐CaP/siCA9 NPs were more beneficial in repolarizing M1 macrophages into the M2 phenotype and promoting chondrogenesis, based on the results of in vivo PCR and immunohistochemistry, which suggested that the NPs might be more advantageous for long‐term therapy in OA.

### 2.8 Inhibitory Effects of NPs on Cartilage Damage and Synovitis in Advanced OA Mice

**Figure 9:**
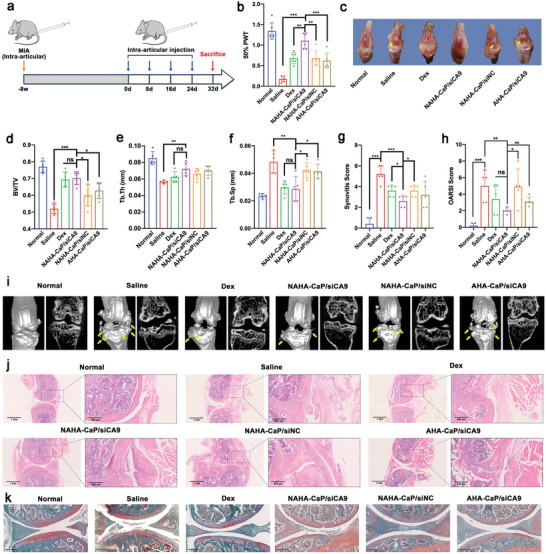
a) Schematic illustration of the experimental design in C57BL/6 mice with advanced OA. b) The paw withdrawal threshold evaluated by von Frey assay at the end of treatment (*n* = 6). c) Representative images of the macroscopic observation of knee joints isolated from mice. The yellow arrows point to scratches or breakages. d) The quantitative measurements of bone volume fraction (BV/TV), e) trabecular thickness (Tb.Th), and f) trabecular separation (Tb.Sp) detected using microCT (*n* = 5). g) Synovitis score of H&E‐stained sections (*n* = 5). h) Cartilage OARSI score in Safranin O‐fast green‐stained sections (*n* = 5). i) The representative microCT bone remodeling 3D and microCT 2D images of the mice knee joints. j) H&E staining images of knee joint tissues isolated from mice with advanced OA. Scale bars were 1 mm and 250 µm, respectively. k) Safranin O‐fast green staining images of mice knee joint tissues with advanced OA. Scale bars, 250 µm. Data are represented as mean ± SD. *p < 0.05, **p < 0.01, ***p < 0.001.

Given the promising therapeutic efficacy of NPs in early‐stage OA, the effects of NAHA‐CaP/siCA9 NPs on advanced OA were further assessed in C57BL/6 mice. Eight weeks after a single intra‐articular injection of 10% (w/v) MIA, an advanced OA mouse model was established. The mice were randomly divided into five groups and treated with different formulations at a siRNA dose of 0.5 mg kg^−1^ via intra‐articular administration for a total of four injections (**Figure** **9**a).

Before treatment, the retention time of Cy7‐siRNA loaded NPs in OA joints through intra‐articular injection was detected using an in vivo imaging system. As shown in Figure S14 (Supporting Information), the fluorescence intensity of the free Cy7‐siRNA gradually decayed and finally disappeared on day 5. Strong fluorescence intensity was observed at the knee joints and remained for up to nine days following injection of NAHA‐CaP/Cy7‐siRNA NPs, which suggested the protective effects of NPs on siRNA and extended retention of NPs in OA joints.

Intermittent but severe pain is the most common and distressing pain in patients with OA, especially in advanced‐stage OA, in which changes in the knee joint structure and exacerbation of synovitis play important roles. To evaluate the therapeutic effect of NPs on pain attenuation, von Frey hair was applied to the hindfoot of mice to quantify the 50% paw withdrawal threshold (50% PWT) at the end of the treatment. As shown in Figure 9b, compared with the normal group, the mice in the saline group showed a greatly reduced 50% PWT, suggesting mechanical allodynia induced by advanced OA. Following the administration of different formulations, the 50% PWT of the mice increased. Among the different formulations, the NAHA‐CaP/siCA9 group exhibited the highest 50% PWT value. The data demonstrated that NAHA‐CaP/siCA9 NPs treatment effectively relieved joint pain and was even better than the Dex group in advanced OA.

After the experimental treatment, the mice were euthanized on day 32, knee joints were harvested, and the results of macroscopic observations, microCT arthrography, and histological staining were collected. As shown in Figure 9c, compared to the normal group, the saline group exhibited severe cartilage erosion. After administration of NAHA‐CaP/siCA9 NPs, reduced lesions and smooth surfaces were observed, indicating ameliorated cartilage destruction caused by OA. MicroCT arthrography showed that compared with normal joints, severe bone defects and apparent osteophytes were observed, accompanied by an unsmooth surface in saline‐treated mice (Figure 9i). At the same time, the quantitative data of the saline group demonstrated a significant reduction in BV/TV and Tb.Th (trabecular thickness, whose decrease indicates OA development) and an increase in Tb.Sp (referring to the average width of the medullary cavity between the trabecular bone, whose increase indicates increased bone resorption) (Figure 9d–f), indicating that severe cartilage degradation and osteoporosis may have occurred. However, markedly fewer osteophytes and much smaller bone defects were observed after the NAHA‐CaP/siCA9 NPs treatment. Remarkably, the NAHA‐CaP/siCA9 group displayed a higher BV/TV and Tb.Th, as well as a lower Tb.Sp value, closer to those of the normal group, demonstrating great improvement in advanced OA therapy by NAHA‐CaP/siCA9 NPs. NAHA‐CaP/siNC and AHA‐CaP/siCA9 showed moderate efficacy in reducing bone erosion.

H&E and safranin O‐fast green staining were performed to observe synovitis and articular cartilage damage (Figure 9j,k). In healthy mouse knee joints, the surfaces of the normal femur and tibia cartilage were intact, ordered, and smooth, and proteoglycans were evenly distributed in the ECM with strong safranin O staining. However, H&E‐stained sections from the saline group showed severe synovial hyperplasia with increased immune cells, and the cartilage of this group was irregular and damaged. In addition, the cartilage of the saline group also showed apparent vertical clefts and erosion along with extensive loss of safranin O staining, suggesting bone and cartilage destruction by the progression of OA. In contrast, the NAHA‐CaP/siCA9 group showed reduced synovial inflammation and decreased loss of cartilage, in which almost no synovial hyperplasia, relatively intact surface of femur and tibia articular cartilage, and strong Safranin O staining was observed. Notably, the NAHA‐CaP/siCA9 group presented more intense Safranin O staining than the Dex group, indicating that NAHA‐CaP/siCA9 NPs could effectively protect articular cartilage in terms of glycosaminoglycan (GAG) deposition, retention of cartilage thickness, and attenuation of cartilage matrix depletion. Furthermore, the notable therapeutic effects of NAHA‐CaP/siCA9 NPs on inhibiting synovial inflammation and degradation of articular cartilage in advanced OA were further evidenced by the dramatically decreased synovitis and OARSI scores, respectively, compared with the saline group (Figure 9g,h).

Overall, the above results indicate that the high efficacy of NAHA‐CaP/siCA9 NPs in anti‐synovial inflammation and cartilage protection, which was achieved through the synergetic effects of NO scavenging and CA9 gene silencing, could mitigate both early and late OA progression.

### 2.9 Inhibitory Effects of NPs on Cartilage Damage and Synovitis in DMM‐Induced OA in Rats

The therapeutic efficacy of NP treatment was evaluated in rats with DMM surgery‐induced OA (Figure S15a, Supporting Information). In saline‐treated rats, mechanical allodynia was observed in the operated hind paws. NAHA‐CaP/siCA9 NP treatment increased the paw withdrawal threshold, which was similar to that of the Dex group, demonstrating that these NPs could relieve OA‐related pain in rats (Figure S15b, Supporting Information).

MicroCT and histological analysis (Figure S15d–f, h,i, and k, Supporting Information) revealed cartilage damage with a high OARSI score in the saline‐treated group, including surface fibrillation and loss of proteoglycan at the end of the treatment, as well as reduced BV/TV and Tb.Th, and increased Tb.Sp. Treatment with NAHA‐CaP/siCA9 NPs improved the morphology and structure of the articular cartilage, leading to an almost intact cartilage surface with no proteoglycan loss. Furthermore, decreased osteophyte formation was observed in the NAHA‐CaP/siCA9 NP‐treated group as compared with that observed in the saline‐treated group. These results indicate that NPs can efficiently prevent OA progression.

Synovial thickness is an indicator of inflammation in the knee joint. Saline‐treated DMM knees exhibited a significantly thickened synovial lining with a 14‐fold increase in synovitis score compared with the sham group. In contrast, the NAHA‐CaP/siCA9 NP‐treated group showed only a fivefold increase (Figure S15g,j, Supporting Information), suggesting that these NPs had anti‐inflammatory effects in the injured knees.

Immunohistochemistry images showed that the expression level of CA9 protein in the saline group was significantly higher than that in the sham group (Figure S16, Supporting Information), demonstrating that increased expression of CA9 was induced in OA joints. NAHA‐CaP/siCA9 NP treatment significantly downregulated CA9 protein expression, which was consistent with the results in OA mouse models.

In addition, no significant body weight loss in rats was observed following treatment with the different formulations, suggesting the biosafety of NPs in rats (Figure S15c, Supporting Information).

### 2.10 In Vivo Safety of NAHA‐CaP/siCA9 NPs

The in vivo biosafety of NAHA‐CaP/siCA9 NPs was evaluated by monitoring changes in body weight, important hematological indicators (HGB, WBC, RBC, and PLT), and biochemical parameters (AST, ALT, UA, UREA, and CREA) in mice with early‐stage OA during the administration period. H&E staining was used to evaluate histopathology of the major organs. As shown in Figure S17a (Supporting Information), no difference in body weight was observed in mice treated with the different formulations during the entire administration period. Moreover, no significant changes in RBC, HGB, WBC, and PLT levels were observed among the groups after treatment (Figure S17b–e, Supporting Information), indicating that these NPs did not induce systemic toxicity and blood dysfunction in mice. Furthermore, the levels of ALT, AST, CREA, UA, and UREA in the mice treated with different NPs were similar to those in the saline group (Figure S17f–j, Supporting Information). In addition, H&E staining revealed that, compared with the saline group, NP‐treated groups did not show pathological abnormalities in major organs (heart, liver, spleen, lung, and kidney), suggesting that these NPs would not induce significant toxicity to normal organs (Figure S17k, Supporting Information). These results demonstrate that NAHA‐CaP/siCA9 NPs are a promising and safe delivery system for in vivo OA therapy.

In vivo therapeutic effects and safety evaluation indicated that NAHA‐CaP/siCA9 NPs could efficiently inhibit synovial inflammation and cartilage destruction based on the synergistic biological activities of siCA9 and NO scavenger, showing biosafety in both MIA‐induced early or late OA mouse model and surgery‐induced OA rat model after intra‐articular administration.

## 3 Conclusion

To the best of our knowledge, this was the first study to demonstrate the significantly increased expression of CA9 in the synovium macrophages of joint tissues from patients with OA and MIA‐induced OA mice. In this study, we successfully constructed a NAHA‐CaP/siCA9 nanocarrier for codelivering siCA9 and NO scavenger with highly efficient and safe OA therapy. After HA‐mediated cell internalization, NAHA‐CaP/siCA9 NPs efficiently downregulated CA9 gene expression and consumed high levels of intracellular NO in LPS‐activated M1 macrophages. NAHA‐CaP/siCA9 NPs suppressed the production of proinflammatory cytokines and inhibited the activation of p38 MAPK, NF‐kB (p50/p65), and MyD88 signaling pathways. Moreover, these NPs promoted macrophage repolarization from the M1 to M2 phenotype, thereby upregulating pro‐chondrogenic cytokines and cartilage matrix‐related gene expression and decreasing macrophage CM‐stimulated chondrocyte apoptosis. The in vivo results also demonstrated that NAHA‐CaP/siCA9 NPs effectively suppressed both synovial inflammation and articular cartilage degeneration in early and advanced OA. More importantly, these NPs had comparable anti‐inflammatory effects to the commercial drug Dex but showed stronger expression of macrophage polarization and cartilage matrix component production‐associated genes, indicating better potential in cartilage protection and repair effects. Therefore, our study provides an alternative strategy with an innovative target for OA therapy based on siCA9 and NO scavenger codelivered NPs, which holds promising application prospects for clinical translation.

## 4 Experimental Section

### Materials

Guanidine hydrochloride were purchased from Sigma Aldrich Co. (St. Louis, MO); (Boc)_2_O, NHS, EDCI, and methyl 3,4‐diaminobenzoate were purchased from Bide Pharmatech Ltd. (Shanghai, China); hyaluronic acid (HA, Mw: 780 kDa) was obtained from Shan Dong Li Young Biotechnology Co., Ltd. (Shangdong, China); Hoechst 33342, Cell Counting Kit‐8 (CCK8), BCA Protein Concentration Assay Kit, Annexin V‐FITC/PI cell apoptosis detection Kit, dialysis bags, alendronate sodium, and Dex were purchased from Solarbio Technology Co., Ltd. (Beijing, China); Lipofectamine 2000, LysoTracker Red DND‐99, and Rhodamine phalloidin were purchased from Invitrogen (NY, USA); CA9 siRNA (siCA9), negative control siRNA (siNC), and fluorescent‐labeled siRNA (5′ end of the sense strand, Cy5‐siRNA) were synthesized and purified with HPLC by RiboBio Co. Ltd. (Guangzhou, China); Opti‐MEM and DMEM medium were purchased from Macgene Technology Co., Ltd. (Beijing, China); lipopolysaccharides from *Escherichia coli* O111:B4 (LPS) was purchased from Sigma (USA); Anti‐CA9 antibody (ab243660) was purchased from Abcam (Shanghai, China); Anti‐*β*‐actin antibody was purchased from EasyBio Technology Co., Ltd. (Beijing, China); Anti‐NF‐*κ*B p65, Anti‐phospho‐NF‐*κ*B p65(Ser536), Anti‐NF‐*κ*B p105/p50, Anti‐P38 MAPK, Anti‐phospho‐P38 MAPK(Thr180+Tyr182), Anti‐STAT6, Anti‐phospho‐STAT6(Tyr641), and Anti‐MyD88 antibody were purchased from Biosynthesis Biotechnology Co., Ltd. (Beijing, China); NF‐*κ*B p65, NF‐*κ*B p50, IL‐6, IL‐10, IL‐1*β*, and TNF‐*α* ELISA kit were purchased from Winter Song Boye Biotechnology Co. Ltd. (Beijing, China); purified Anti‐Mouse CD16/32 Antibody[2.4G2], PE Anti‐Mouse CD206 Antibody[C068C2], APC Anti‐Mouse CD86 Antibody[GL‐1], PerCP/Cyanine5.5 Anti‐Mouse F4/80 Antibody[CI:A3‐1], and CA9 ELISA kit were purchased from Elabscience Biotechnology Co., Ltd. (Wuhan, China); MIA was obtained from Adamas (Shanghai, China); Griess Reagent kit, DAF‐FM DA, and Caspase 3 activity detection kit were purchased from Beyotime Biotechnology Co., Ltd. (Shanghai, China).

### Patient Samples/Ethics

Human synovial tissue sections were obtained from patients with OA in Peking University Third Hospital who underwent knee replacement surgery or arthroscopy. All patients met the American College of Rheumatology 2009 criteria for RA and 1995 criteria for OA with their agreements to participate in this study, and the studies were carried in accordance with ethics committee approval obtained from Peking University Third Hospital (No. 2013003(2)). Informed written consent of all participants were obtained.

### Cell Culture

RAW264.7 murine macrophages were obtained from Institute of Basic Medical Science, Chinese Academy of Medical Sciences (Beijing, China). The cells were cultured in Dulbecco's modified Eagle medium (DMEM) supplemented with 10% fetal bovine serum (PAN, Germany), 100 U mL^−1^ penicillin, and 0.1 mg mL^−1^ streptomycin (Macgene, Beijing, China). Cells were cultured in a humidified incubator with 5% CO_2_ at 37 °C. The cells for all experiments were in the logarithmic phase of growth.

Notably, in this study the “normal macrophages” refers to the RAW264.7 macrophage cells without LPS treatment (M0 macrophages), and the “activated macrophages” refers to the RAW264.7 macrophage cells treated with LPS (5 µg mL^−1^) for 24 h (M1 macrophages).

Chondrogenic cell ATDC5 was cultured with DMEM (Gibco, USA), containing 100 U mL^−1^ penicillin, 100 mg mL^−1^ streptomycin sulfate, and 10% FBS in a humidified incubator with 5% CO_2_ at 37 °C.

### Primary Chondrocytes Extraction and Culture

Male C57BL/6 mice were sacrificed and disinfected in 75% alcohol for 10 min. The femur head was exposed under aseptic conditions, and the total articular cartilage was further isolated, collected and cut into small fragments (<1 mm^3^ pieces). Then the cartilage tissues were washed thoroughly with sterile PBS for three times and digested with 0.2% type II collagenase for 8–12 h at 37 °C. The released chondrocytes were then washed with sterile PBS by three times. Afterward, the collected chondrocytes were cultured in the DMEM supplemented with 10% FBS and 1% antibiotics in an incubator (37 °C and 5% CO_2_). Culture medium was changed every 2–3 d. When reaching at 70%–80% confluency, chondrocytes were harvested by trypsinization with 0.05% trypsin‐EDTA and split as 1:3 to a new culture flask. The cells at passages 2–3 were used in the following experiments.

### Animals

6–8 weeks male C57BL/6 mice and Sprague‐Dawley (SD) male rats (6–8 weeks) were purchased from Beijing Vital River Laboratory Animal Technology Company Limited and kept in the specific pathogen‐free (SPF) environment with 12‐h light/dark cycle and unrestricted food and water intake were guaranteed. All animal care and experiments were in compliance with the approval of Institutional Authority for Laboratory Animal Care of Peking University (Approval No. LA2021499).

### Synthesis of Materials

*Synthesis of AHA*: NHS (0.126 g, 1.1 mmol) and EDCI (0.210 g, 1.1 mmol) were added sequentially to a solution of hyaluronic acid (0.403 g, 5.2 µmol) (780 kDa) and alendronate sodium (0.126 g, 1.1 mmol) in ultrapure water at room temperature. The resulting mixture was stirred for 48 h and then dialyzed (5000 Da) in ultrapure water overnight. The product (AHA), as a white powder, was obtained through vacuum drying.

*Synthesis of S1*: (Boc)_2_O (2.180 g, 10.0 mmol) and guanidine hydrochloride (0.090 g, 2.0 mmol) were added sequentially to a solution of methyl 3,4‐diaminobenzoate (1.660 g, 10.0 mmol) in alcohol. The resulting solution was stirred at 45 °C for 2 h, at which point TLC analysis (PE/EA = 2:1) indicated the disappearance of starting material. The reaction mixture was concentrated and the residue was purified by column chromatography to afford the product (S1) as a yellow powder.

*Synthesis of S2*: NHS (0.210 g, 1.1 mmol) and EDCI (0.126 g, 1.1 mmol) were added sequentially to a solution of AHA (0.492 g, 5.2 µmol) and S1 (0.492 g, 1.8 mmol) in ultrapure water at room temperature. The resulting mixture was stirred for 48 h and then dialyzed (5000 Da) in ultrapure water overnight. The resulting mixture was stirred for 48 h and then dialyzed (5000 Da) in ultrapure water overnight. The product (S2), as a grey powder, was obtained through vacuum drying.

*Synthesis of NAHA*: 5 mL HCl/EA (2.0 m) was added to a solution of S2 (0.500 g, 4.9 µmol) in EA at room temperature. The resulting mixture was stirred for 4 h. The product (NAHA), as a grey powder, was obtained through vacuum drying.

### Preparation and Characterization of NPs

NAHA‐CaP/siRNA nanoparticles were prepared using nanoprecipitated methods. In brief, 20 µL of siRNA (5 × 10^−6^ m) and 20 µL of CaCl_2_ (500 × 10^−6^ m) were mixed as solution A. 20 µL of HBS (50 × 10^−3^ m HEPES, 280 × 10^−3^ m NaCl, and 1.5 × 10^−3^ m Na_2_HPO_4_, pH = 7.4) and 60 µL of NAHA solution (10 × 10^−6^ m) were mixed as Solution B, then Solution B was added dropwise to Solution A. Similarly, for preparation of AHA‐CaP/siRNA NPs, 20 µL of siRNA (5 × 10^−6^ m) and 20 µL of CaCl_2_ (400 × 10^−3^ m) were mixed as Solution A. 20 µL of HBS (50 × 10^−3^ m HEPES, 280 × 10^−3^ m NaCl, and 1.5 × 10^−3^ m Na_2_HPO_4_, pH = 7.4) and 60 µL of AHA solution (1 × 10^−6^ m) were mixed as Solution B, then Solution B was added dropwise to Solution A.

The particle size and zeta potential of different NPs were measured using DLS (Malvern Zetasizer Nano ZS, Malvern, UK) at 25 °C and at a scattering angle 90°. In addition, the morphology was observed using TEM (JEM‐1400 plus), SEM (JSM‐7900F), and AFM (Dimension Icon). For storage stability assay, the prepared nanoparticles were placed at 4 °C for 1, 2, 3, 5, 7, and 9 d, respectively, and the average particle size were measured using DLS. For dilution stability assay, the prepared nanoparticles were diluted by 2, 4, 8, 16, 32, 64, and 80 times in HBS buffer, respectively, and the size were measured using DLS.

The encapsulation efficiency of siRNA in the NAHA‐CaP/siCA9 NPs was evaluated using a Quant‐iT Ribogreen assay kit (ThermoFisher, USA). Assay solutions were prepared based on manufacturer's instructions, and Ribogreen fluorescence was measured using the high range assay. The NPs solutions were prepared at 1 × 10^−6^ m siRNA, and 10 µL of NPs solution was diluted by 90 µL in 1× TE buffer, followed by addition of 100 µL Ribogreen reagent to each well. After 5 min of mixing, the fluorescence intensity of samples was measured using a fluorescence microplate reader at the excitation wavelength of 485 nm and emission wavelength of 525 nm. The encapsulation efficiency was the ratio of encapsulated siRNA to a total siRNA.

### Gel Retardation Assay

To evaluate the protective effects on siRNAs, the nanoparticles were mixed with FBS at the volume ratio of 1:1 and then incubated at 37 °C for different time. Then the samples were mixed with 6× loading buffer containing 1.0 m HCl and electrophoresis was carried out on a 1% agarose gel containing 0.1% GelRed at 80 mV for 3 min, subsequently 100 mV for 25 min, and these resulting gels were observed using an Amersham Imager 600 imaging system. Free siRNAs were used as the control.

### pH‐Responsive Disassembly of NPs

The prepared nanoparticles were diluted in HEPES buffer with different pH values (7.4, 6.8, 6.2, 5.6, and 5.0) and incubated for 1 h at room temperature. Then the particle size was monitored using Malvern dynamic light scattering particle size analyzer. And the morphologies of NPs were also observed using SEM.

### pH‐Responsive Release of siRNA from NAHA‐CaP/siRNA NPs

The prepared Cy5‐siRNA‐loaded nanoparticles were placed in a dialysis bag (MWCO = 20 kDa), and dialyzed with HBS solution (V/V = 1/20) at different pH (7.4 or 5.0). At the predetermined time points (3 min, 5 min, 10 min, 20 min, 30 min, 1 h, 2 h, 6 h, 12 h, 24 h, and 48 h), the fluorescence intensity of released Cy5‐siRNA was detected using a fluorescence microplate reader (Varioskan LUX) at the excitation wavelength of 650 nm and emission wavelength of 670 nm. Cumulative release rate of siRNA = (*A*_sample_ − *A*_0_)/(*A*_positive_ − *A*_0_) × 100%. *A*_0_ means fluorescence intensity of dialysate at the beginning of dialysis, and *A*_positive_ represents the fluorescence intensity of total Cy5‐siRNA completely released from nanoparticles.

### In Vitro NO‐Scavenging Ability of NAHA‐CaP/siRNA NPs

The preparation of NO and its stock solutions was carried out according to the reported methods.^[^ ^37^ ^]^ NO could be generated by slowly dropping 2.0 m H_2_SO_4_ (aq) into a glass flask containing a saturated NaNO_2_ water solution. Since O_2_ would rapidly oxidize NO to form NO_2_, all apparatus were carefully degassed with argon for 30 min to remove O_2_. The forming gas was passed through a 30% NaOH to trap NO_2_ generated from the reaction of NO with traces of O_2_. To produce a saturated NO solution (1.8 × 10^−3^ m, at 20 °C) as a stock solution, 10 mL of deoxygenated deionized water was bubbled with NO for 30 min. The saturation concentration was ascertained by using the Griess assay. All the NO solutions were freshly prepared just before the experiments.

For the confirmation of NO‐responsiveness of NAHA detected using ^1^H NMR, NAHA material (5 mg) was dissolved in 2.0 mL distilled water and bubbled with freshly prepared NO for 30 min. After dialysis treatment, aqueous solution was freeze‐dried to obtain solid powder. And then the resulting solid powder was dissolved in DMSO for NMR measurement.

For the measurement of NO‐scavenging ability of NAHA‐CaP/siRNA NPs, 50 µL NO sample solution was added into 300 µL NAHA‐CaP/siRNA NPs. After incubation of 30 min at room temperature, the remained NO was measured using Griess assay following the protocol of the manufacturer.

The extracellular levels of NO scavenged by NAHA‐CaP/siRNA NPs were determined using Griess assay. RAW264.7 cells were seeded on 12‐well plate at a density of 3.5 × 10^5^ cells per well under DMEM and incubated for overnight. Medium was replaced by 1 mL of fresh medium containing 5 µg mL^−1^ of LPS. After incubation of 24 h, the cells were treated with different formulations in DMEM medium (the concentration of siRNA was 100 × 10^−9^ m) for another 12 or 24 h. The medium was collected, centrifuged at 1000 *g* for 5 min to discard the cell debris, and the supernatant was used for quantification of whole nitrite by Griess reagent kit (Beyotime Biotechnology) according to the instructions.

For intracellular NO levels determination, the above cells were washed by three times in PBS, and lysed in 200 µL of cell lysate (Beyotime Biotechnology), then collected after centrifuge at 12 000 *g* for 5 min. The supernatant medium was collected and used the Griess kit to measure the intracellular NO content.

In addition, intracellular NO concentration was also detected using the probe DAF‐FM DA (Beyotime Inst. Biotech, Haimen, China) according to the manufacturer's protocol. In brief, RAW264.7 cells were seeded into 12‐well plates (3.5 × 10^5^ cells per well) and cultured for 24 h. After treated with LPS for 24 h, the cells were continued to incubate in DMEM containing different siRNA‐loaded (100 × 10^−9^ m) formulations for 12 or 24 h. Then 5 × 10^−6^ m of DAF‐FM DA were added to incubate for 20 min. For flow cytometer assay, the cells were digested with trypsin and centrifuged at 2000 rpm for 3 min. The cells were collected and resuspended in 400 µL of PBS. Intracellular DAF‐FM DA fluorescence intensity was measured with excitation and emission wavelengths of 495 and 515 nm, respectively. For CLSM assay, the cells were washed with cold PBS for three times and fixed with 4% paraformaldehyde for 15 min at room temperature. Afterward, the cells were stained with Hoechst 33342 (10 µg mL^−1^) for 10 min, washed with PBS for three times, and sealed with 1 mL of glycerol/PBS (V/V = 9/1). Finally, the cells were visualized under a Zeiss LSM880 confocal fluorescence microscope.

### In Vitro Cell Cytotoxicity Assay

CCK‐8 (Donjindo, Japan) assay was used to evaluate cell proliferation and cytotoxicity. RAW264.7 cells or primary chondrocytes of mice (8.0 × 10^3^ cells per well) were seeded into a 96‐well plate and incubated in the incubator at 37 °C with 5% CO_2_. After culturing for 24 h, the cells were treated with different formulations in DMEM medium for another 24 or 48 h (the concentration of siRNA was 100 × 10^−9^ m). Afterward, 10 µL of CCK‐8 solution was added into each well of the plate, and then the cells were cultured for another 2 h. The absorbance of the solution was measured using a microplate reader (Varioskan LUX) at a wavelength of 450 nm.

### In Vitro Cellular Uptake

For flow cytometry, RAW264.7 cells (3.0 × 10^5^ cells per well) were seeded in 12‐well plates and incubated for 24 h. Then the cells were treated with LPS (5 µg mL^−1^) for 24 h. After that, the cells were incubated with different formulations containing Cy5‐siRNA at the final concentration of 100 × 10^−9^ m for 6 h at 37 °C. The cells were washed with cold PBS, treated with trypsin, collected by centrifugation, and resuspended in PBS. The intracellular Cy5 fluorescence intensity (1 × 10^4^ cells) was detected using a FACS Calibur flow cytometry (Becton Dickinson, San Jose, CA) immediately.

For CLSM, RAW264.7 cells were seeded in confocal dishes (20 mm) at the density of 4 × 10^5^ cells per well and incubated for 24 h. The cells were treated with LPS (5 µg mL^−1^) for 24 h and then incubated with Cy5‐siRNA‐loaded formulations for 6 h at 37 °C. After that, the cells were washed with cold PBS for three times and fixed with 4% paraformaldehyde for 15 min at room temperature. Afterward, the cells were stained with Hoechst 33342 (10 µg mL^−1^) and Rhodamine‐labeled phalloidin (200 × 10^−9^ m) for 10 min. Cells were visualized under a Zeiss LSM880 confocal fluorescence microscope.

### Endosomal/Lysosomal Escape Ability

RAW264.7 cells (4 × 10^5^) were seeded in confocal dishes (20 mm) and incubated for 24 h. The cells were treated with LPS (5 µg mL^−1^) for 24 h and then incubated with Cy5‐siRNA‐loaded formulations for 4, 6, and 8 h, respectively. LysoTracker Red (Invitrogen, Carlsbad, USA) (300 × 10^−9^ m) and Hoechst 33342 (Solarbio, Beijing, China) (10 µg mL^−1^) were added to the medium before the end of incubation. After staining for 30 min, the cells were fixed by 4% paraformaldehyde and observed under Zeiss LSM880 confocal fluorescence microscope.

### In Vitro Real Time‐PCR Assay

RAW264.7 cells (4 × 10^5^ cells per well) were seeded into six‐well plates and cultured for 24 h. The cells were treated with LPS (5 µg mL^−1^) overnight and then incubated with different formulations containing siCA9 or siNC in Opti‐MEM at a final concentration of 25, 50, 75, and 100 × 10^−9^ m, respectively for 6 h. After that, the Opti‐MEM medium was replaced with fresh complete medium and incubated for another 24 h. Total RNA of the cells was extracted using TRIZOL reagent (Invitrogen). RNA concentration and purity were determined via measuring the absorbance at 260 and 280 nm, respectively. The Reverse Transcription Kit GoScript Reverse Transcription System (#A5001, Promega, USA) was used to reversely transcribe the RNA, GoTaq qPCR Master Mix (#A6002, Promega, USA) was used to amplify the cDNA. RT‐PCR was performed on a Real‐Time PCR amplifier (CFX Connect, Bio‐Rad, USA). The mRNA expression levels were expressed by 2^−(Ct−Cc)^ (Ct and Cc are the mean threshold cycle differences after normalizing to *β*‐actin).

### Western Blotting Analysis

Western blotting assay was performed to evaluate the expression levels of proteins. RAW264.7 cells (5 × 10^5^ cells per well) were seeded into six‐well plates and cultured for 24 h. The cells were treated with LPS (5 µg mL^−1^) overnight and then incubated with different formulations containing siCA9 or siNC in Opti‐MEM at a final concentration of 100 × 10^−9^ m for 6 h. After that, the Opti‐MEM medium was replaced with fresh complete medium and incubated for another 24 h. Afterward, the cells were washed twice with PBS and lysed in 200 µL RIPA lysis buffer supplemented with 1% proteinase inhibitor cocktail on ice for 30 min. The total protein was gathered by centrifugation at 12 000 *g* for 15 min and the concentration of protein was determined by BCA protein assay kit (Solarbio, Beijing, China). A 40 µg amount of total protein of each sample was loaded and separated by 10% sodium dodecyl sulfate polyacrylamide gel electrophoresis (SDS‐PAGE). After transferring polyvinylidene difluoride (PVDF) membranes and blocking with 5% nonfat skimmed milk, the membranes were incubated with various primary antibodies, followed by detection with horseradish peroxidase (HRP)‐linked IgG secondary antibodies. Finally, bands were visualized by enhanced chemiluminescence, and imaged using Amersham Imager 600 system (GE Healthcare Life sciences, USA). *β*‐actin was used as endogenous control.

### Measurement of Inflammation Factors Levels Using ELISA Kit

RAW264.7 cells (3 × 10^5^ cells per well) were seeded into 12‐well plates and cultured for 24 h. The cells were treated with LPS (5 µg mL^−1^) overnight to induce activated macrophages, then treated with different formulations (the concentration of siRNA was 100 × 10^−9^ m) containing DMEM for another 24 h. For measurement of the extracellular pro‐inflammation factors secreted from cells, the supernatants were collected and the levels of TNF‐*α*, IL‐1*β*, and IL‐6 were measured using their corresponding ELISA kits according to the manufacture's protocol. Normal macrophages and activated macrophages without only treatment were served as NT and control group, respectively. For evaluation of intracellular pro‐inflammatory cytokine levels, the treated cells were washed three times with PBS and lysed in 200 µL RIPA lysis buffer supplemented with 1% proteinase inhibitor cocktail on ice for 30 min. After that, the cells were collected by centrifuging at 4 °C and 12 000 rpm for 15 min. The protein supernatant was used for ELISA assay according to the manual.

### Macrophage Repolarization from M1 to M2 Phenotype Analysis

To assess macrophage repolarization from M1 to M2, RAW264.7 macrophages were stimulated with LPS for 24 h to display the M1 phenotype. At the same time, the M1 macrophages were treated with various formulations for additional 24 h (the siRNA concentration was 100 × 10^−9^ m). Subsequently, the polarization transitions were evaluated using qRT‐PCR and flow cytometry analysis. For RT‐PCR assay, the expression of M1 marker (IL‐12 and iNOS) and M2 marker (IL‐10, CD206, Arg‐1, and TGF‐*β*1) mRNA were detected.

For flow cytometry analysis, CD86 was chosen to mark the M1 phenotype and CD206 for the M2 phenotype. PE Anti‐Mouse CD206 Antibody and APC Anti‐Mouse CD86 Antibody (Elabscience) were used to evaluate macrophage subsets. Samples were further analyzed using a FACScan flow cytometer (BD).

### Preparation of CM

To investigate the pro‐chondrogenic effect of NP‐treated macrophages on ATDC5 cells, CM of macrophages was prepared and used for culturing ATDC5 cells. Briefly, RAW264.7 macrophage cells (5 × 10^5^ cells per well) were seeded onto 12‐well plates and cultured for 24 h. The cells were treated with LPS (5 µg mL^−1^) and various formulations in DMEM medium (the concentration of siRNA was 100 × 10^−9^ m) for another 24 h. The NT (not treated) group represented macrophage CM treated with DMEM complete medium, the control group represented macrophage CM induced with LPS. Different CMs were acquired by filtering cell‐free supernatants through a 0.22 mm filter unit.

### Chondrogenesis Ability of CM on ATDC5 Cells

ATDC5 cells (3 × 10^5^ cells per well) were seeded onto 12‐well plates and cultured for 24 h. The obtained different CMs were used to culture normal ATDC5 cells for another 48 h. After that, the qRT‐PCR assay was used to investigate the expression of chondrogenesis‐related genes (such as Col2*α*1 and Acan).

### Analysis of Caspase 3 Activity

ATDC5 cells (4 × 10^5^ cells per well) were seeded in six‐well plates and incubated for 24 h, then treated with various CMs and incubated for another 48 h at 37 °C. After that, the cells were washed by PBS and collected by centrifuging at 600 *g* for 5 min, suspended in cell lysis buffer, and incubated on ice for 30 min. After centrifugation at 16 000 *g* for 10 min at 4 °C, the supernatant was collected and stored for measurement. The activity of Caspase 3 was detected using Caspase 3 Activity Assay Kit (Beyotime Inst. Biotech, Haimen, China) according to the introduction. Briefly, for each well in a 96‐well microplate, samples (50 µL), assay buffer (40 µL), and Caspase 3 substrate (Ac‐DEVD‐*p*NA, 10 µL) were mixed. The samples were then incubated at 37 °C for 4 h and measured with a microplate reader at a wavelength of 405 nm. The Caspase 3 activity was exhibited as fold of the values obtained for the untreated control cells.

### In Vitro Cell Apoptosis Analysis

ATDC5 cells (4 × 10^5^ cells per well) were seeded into six‐well plates and cultured for 24 h, then treated with different CMs for another 48 h. Then the cells were digested by trypsin, collected by centrifugation at 2000 rpm for 5 min, and suspended in 500 µL of 1× binding buffer. The cells were mixed with 5 µL of Annexin V‐FITC and 5 µL of PI staining solution (Macgene, M&C Gene Technology) and kept in dark for 15 min at room temperature, then detected using flow cytometry. Data were collected and analyzed using CELLQuest software.

### Establishment of Mouse Models with Early‐Stage and Advanced OA

Experimental procedures were conducted under general anesthesia. For early‐stage OA, mice were given a single intra‐articular injection of 1% (w/v) MIA (20 µL). Two weeks after OA induced, the mice were randomly divided into five groups. For advanced OA, mice were given a single intra‐articular injection of 10% (w/v) MIA (20 µL). Eight weeks after OA induced, the mice were randomly divided into five groups. Different formulations were used for OA treatments by intra‐articular injection (20 µL): (1) Normal saline, (2) Dex (1 mg kg^−1^), (3) NAHA‐CaP/siCA9 NPs, (4) NAHA‐CaP/siNC NPs, and (5) AHA‐CaP/siCA9 NPs. Healthy mice were used as the control group. The siRNA dose was 0.5 mg kg^−1^ and the dosing interval was eight days.

### Establishment of DMM‐Induced OA in Rats

To induce OA that mimics chronic OA in human patients, male rats (*n* = 30) at ten weeks of age were subjected to DMM surgery at right knees followed by the operations described in the literature.^[^ ^38^ ^]^ Briefly, each rat was allowed to acclimate to the environment for one week before surgery. After anesthesia, the right knee joint capsule was opened immediately with a 3 mm medial capsule incision, followed by gentle lateral displacement of knee extensor muscles. The medial meniscotibial ligament (MMTL) was exposed and cut to destabilize the medial meniscus without damaging other tissues. In sham surgery, the joint capsule was opened in the same fashion but MMTL was not actually cut. After surgery, animals were permitted free activity with sufficient food and water intake provided.

### In Vivo Joint Retention Assay

The mouse knee joints retention assay was assessed by intra‐articular injection of 20 µL of free Cy7‐siRNA or Cy7‐siRNA‐loaded NAHA‐CaP/siRNA NPs (at a siRNA dose of 0.5 mg kg^−1^). At different post injection, the fluorescent images of the mice were imaged using an in vivo imaging system (IVIS SPECTRUM, Spectrum, PerkinElmer) with the excitation wavelength of 745 nm and emission wavelength of 800 nm. All images were normalized and analyzed using Living Image software.

### In Vivo OA Pain Analysis

The knee joint pain of mice (or rat) was evaluated weekly using von Frey filaments (or electronic von Frey aesthesiometer) as described in the literature.^[^ ^39^ ^]^ Briefly, an individual mouse was placed on a wire‐mesh platform under a 4 cm × 3 cm × 7 cm cage to restrict their move. During the test, a set of von Frey fibers (ranging from 0.028‐ to 5.5‐g force for mice) were applied to the plantar surface of the hind paw until the fibers bowed and then held for 3 s. Then recorded the response [foot withdrawal (mark X) or no foot withdrawal (mark 0)], and then repeat, using the next higher successive von Frey filament if there was no response to the previous filament, or the next lower successive von Frey filament if there was a response to the previous filament. The threshold force corresponding to 50% withdrawal was calculated by this up‐down iterative method, which required continuing testing until four stimuli had been applied following the first response reversal with sequential measurements separated by at least 5 min.

### Microcomputed Tomography Analysis

At the end of the treatment, the knee joints of mice were harvested and fixed in 4% neutral formaldehyde for 48 h. The distal femur and proximal tibia were scanned at a 6 µm isotropic voxel size with a microCT scanner (Inveon, Siemens, Erlangen, Germany). The ROI, trabecular bone, and subchondral bone of both tibia and femur were manually circled, and bone morphology parameters including the ratio of bone volume to tissue volume (BV/TV), trabecular separation (Tb.Sp), trabecular thickness (Tb.Th), and trabecular number (Tb.N) were calculated using 3D reconstruction analysis.

### Histological Staining Analysis

For histological analysis, mice and rats were sacrificed after treatment of different formulations, and the OA knee joints were harvested for fixation in 10% formalin for 24 h. Formic acid (10% v/v) was used to decalcify, and paraffin was employed to embed samples. After 4‐µm serial sectioning of the knee joint, H&E and safranin O‐fast green staining were performed to evaluate the changes to the cartilage microstructure as well as severity of synovitis after NP treatment. For semiquantitative analysis of OA progression and inflammation level of synovium, the OARSI score^[^ ^40^ ^]^ and synivitis scoring^[^ ^41^ ^]^ were applied as described previously.

For immunohistochemistry assay, the sections of embed tissues were incubated with primary antibodies (1:200, overnight at 4 °C) and then incubated with biotinylated secondary antibody for 1 h. After washing in PBS, sections were incubated with DAB (Servicebio, Boston, USA) before the reaction was stopped in distilled water and counterstained with hematoxylin. Stained slides were observed using Leica Q550CW imaging system (200×).

To evaluate the systemetic toxicity of nanoparticles in mice after intra‐articular injection, major organs including hearts, livers, spleens, lungs, and renal tissues were dissected and fixed in 4% paraformaldehyde overnight for H&E staining.

### In Vivo Detection of Inflammatory Factors

To determine the levels of IL‐6, IL‐1*β*, and TNF‐*α* in the serum or knee joint tissues of different groups after the treatment, serum samples or joint tissues isolated from mice were collected at the end of treatments. The concentrations of inflammatory factors were determined using ELISA kits (Dogesce) according to the manufacturer's instruction.

### In Vivo Detection of mRNA or Protein Expression

The human or mice tissues were used for detecting mRNA and protein expression levels by qRT‐PCR and immunohistochemistry, respectively. For RT‐PCR assay, the joint tissues ex vivo were ground in Trizol reagent at 4 °C and total RNA was extracted. The other operations were performed as the same as described above.

For immunohistochemistry assay, the knee joints were harvested, fixed in 4% paraformaldehyde, and disposed as above to fix and decalcify. Afterward, the paraffin‐embedded joints were used for assays. Briefly, 5 µm histological sections were immersed in xylene, 95% alcohol, 85% alcohol, and 75% alcohol for 5 min, respectively, and washed with PBS (pH = 7.4) for three times after each immersion. Then the sections were incubated with primary antibodies against CA9, MMP13, Col2*α*1, or CD206 (1:200, overnight at 4 °C) and then incubate with biotinylated secondary antibody for 1 h. After washing in PBS, sections were incubated with DAB (Servicebio, Boston, USA) before the reaction was stopped in distilled water and counterstained with hematoxylin. Stained slides were observed using pathological slide scanner (Buchi).

### In Vivo Biosafety of NPs

The body weight of mice or rats was measured every day. For histology analysis, major organs (heart, liver, spleen, lung, and kidney) were sectioned and stained with H&E, then observed using Digital pathology slide scanner (Buchi). For blood routine test, blood samples were drawn from the venous plexus of the eyes to measure the concentrations of the RBC, WBC, PLT, and hemoglobin at the end of the treatment. For biochemical index analysis, blood samples were collected under non‐anticoagulation condition and centrifuged with 3000 rpm for 10 min at 4 °C. The supernatant was then collected and diluted for use.

### Statistical Analysis

All the results were presented as mean ± standard deviation (SD). Unpaired Student's *t*‐test (two‐tailed) was used for two‐group comparison and one‐way ANOVA followed by Tukey's test was applied for multiple‐group statistical analysis. All the statistical analyses were performed by using GraphPad Prism Software (Version 8.0, GraphPad Software, San Diego, CA). A value of *p* < 0.05 was considered as statistically significant difference.

## Conflict of Interest

The authors declare no conflict of interest.

## Supporting information

Supporting Information

## Data Availability

The data that support the findings of this study are available from the corresponding author upon reasonable request.
