# Supplementary material for: Nanomedicines Reprogram Synovial Macrophages by Scavenging Nitric Oxide and Silencing CA9 in Progressive Osteoarthritis
Source: Adv Sci (Weinh). 2023 Feb 7;10(11):2207490. doi: 10.1002/advs.202207490 (PMC10104675; doi:10.1002/advs.202207490)
Supplement: Supplementary file 1 — Supporting Information [file ADVS-10-2207490-s001.pdf]

WILEY-VCH

ADVANCED
SCIENCE

Supporting Information

Nanomedicines Reprogram Synovial Macrophages by Scavenging Nitric Oxide and Silencing CA9 in Progressive Osteoarthritis

Yi Yan[1#], An Lu[1#], Yun Dou[2#], Zhen Zhang[2], Xiang-Yu Wang[1], Lin Zhai[1], Li-Ya Ai[2], Ming-Ze Du[2], Lin-Xia Jiang[1], Yuan-Jun Zhu[1], Yu-Jie Shi[1], Xiao-Yan Liu[1], Dong Jiang[2] and Jian-Cheng Wang[1,3]**

[1] Beijing Key Laboratory of Molecular Pharmaceutics and New Drug Delivery Systems, State Key Laboratory of Natural and Biomimetic Drugs, School of Pharmaceutical Sciences, Peking University, Beijing, China.

[2] Department of Sports Medicine, Peking University Third Hospital, Beijing, China.

[3] Laboratory of innovative formulations and pharmaceutical excipients, Ningbo Institute of Marine Medicine, Peking University, Beijing, China.

*Corresponding author: Jian-Cheng Wang
Beijing Key Laboratory of Molecular Pharmaceutics and New Drug Delivery Systems, State Key Laboratory of Natural and Biomimetic Drugs, School of Pharmaceutical Sciences, Peking University, XueYuan Rd 38, Haidian Dist, Beijing 100191, China.
E-mail: wang-jc@bjmu.edu.cn; Tel/fax: +86-010-82805932.
*Corresponding author: Dong Jiang
Department of Sports Medicine, Peking University Third Hospital, Beijing, China.
E-mail: bysyjiangdong@126.com; Tel: +86-010-82267020; Fax: +86-010-62010440.

[#] These authors contributed equally to this work.

Keywords: Carbonic anhydrase IX, osteoarthritis, siRNA delivery, nitric oxide, macrophages, polarization, cartilage protection

Tables

Table S1. Characterization of NPs (n = 3).

Nanoparticles	Mean particle size (nm)	Polydispersity Index (PDI)	Zeta potential (mV)
NAHA-CaP/siRNA	188.95 ± 15.48	0.166 ± 0.079	-12.85 ± 4.11
AHA-CaP/siRNA	193.86 ± 9.03	0.272 ± 0.014	-12.10 ± 4.21

Table S2. siRNA sequences used in this study.

siRNA		Sequence
siNC	Sense	5’- UUCUCCGAACGUGUCACGUTT -3’
	Antisense	5’- ACGUGACACGUUCGGAGAATT -3’
siCA9-mice	Sense	5’- CAGUACUGCUUUCUCCGAACUUCAU -3’
	Antisense	5’- AUGAAGUUCGGAGAAAGCAGUACUG -3’
siCA9-rat	Sense	5’-GAGCTGATGTTGACTTGAA -3’
	Antisense	5’-CTCGACTACAACTGAACTT-3’

Table S3. Primers for qRT-PCR.

Gene	Species		Primer
β-actin	Mice	Forward	5’- GGTCATCACTATTGGCAACG -3’
		Reverse	5’- ACGGATGTCAACGTCACACT -3’
IL-12		Forward	5’- CACGGCAGCAGAATAAATA -3’
		Reverse	5’- CTTGAGGGAGAAGTAGGAATG -3’
IL-10		Forward	5’- GCTCTTACTGACTGGCATGAG -3’
		Reverse	5’- CGCAGCTCTAGGAGCATGTG -3’
iNOS		Forward	5’- AGCCAAGCCCTCACCTACTT -3’
		Reverse	5’- CTCTGCCTATCCGTCTCGTC -3’
MMP13		Forward	5’- TGTTTGCAGAGCACTACTTGAA -3’
		Reverse	5’- CAGTCACCTCTAAGCCAAAGAAA -3’
CD206		Forward	5’- GGAGGCTGATTACGAGCAGT -3’
		Reverse	5’- CATAGGAAACGGGAGAACCA -3’
CA9		Forward	5’- TTCAGTCCCCGGTAGACATC -3’
		Reverse	5’- TTTCTTCCAAATGGGACAGC -3’
Col2α1		Forward	5’- TGGTGCTCGGGGTAACGAT -3’
		Reverse	5’- GGCTCCAGGAATACCATCAGT -3’
Acan		Forward	5’- ATTTCCACACGCTACACCCTG -3’
		Reverse	5’- TGGATGGGGTATCTGACTGTC -3’
TGF-β1		Forward	5’-CCACCTGCAAGACCATCGAC-3’
		Reverse	5’-CTGGCGAGCCTTAGTTTGGAC-3’
Arg1		Forward	5’- CTCCAAGCCAAAGTCCTTAGAG -3’
		Reverse	5’- GGAGCTGTCATTAGGGACATCA -3’
GAPDH	Human	Forward	5’- CTGACTTCAACAGCGACACC -3’

	Reverse	5’- TAGCCAAATTCGTTGTCATACC -3’
CA9	Forward	5’- GGGTGTCATCTGGACTGTGTT -3’
	Reverse	5’- CTTCTGTGCTGCCTTCTCATC -3’

Figures

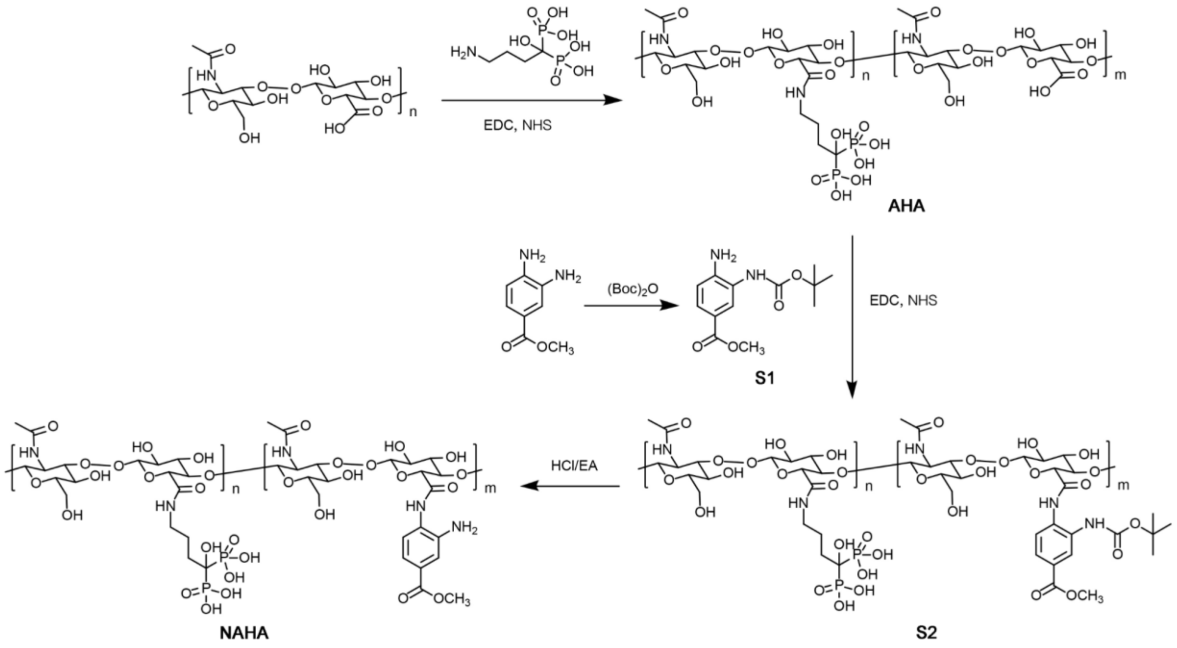

Figure S1. Synthetic route of NAHA and AHA materials.

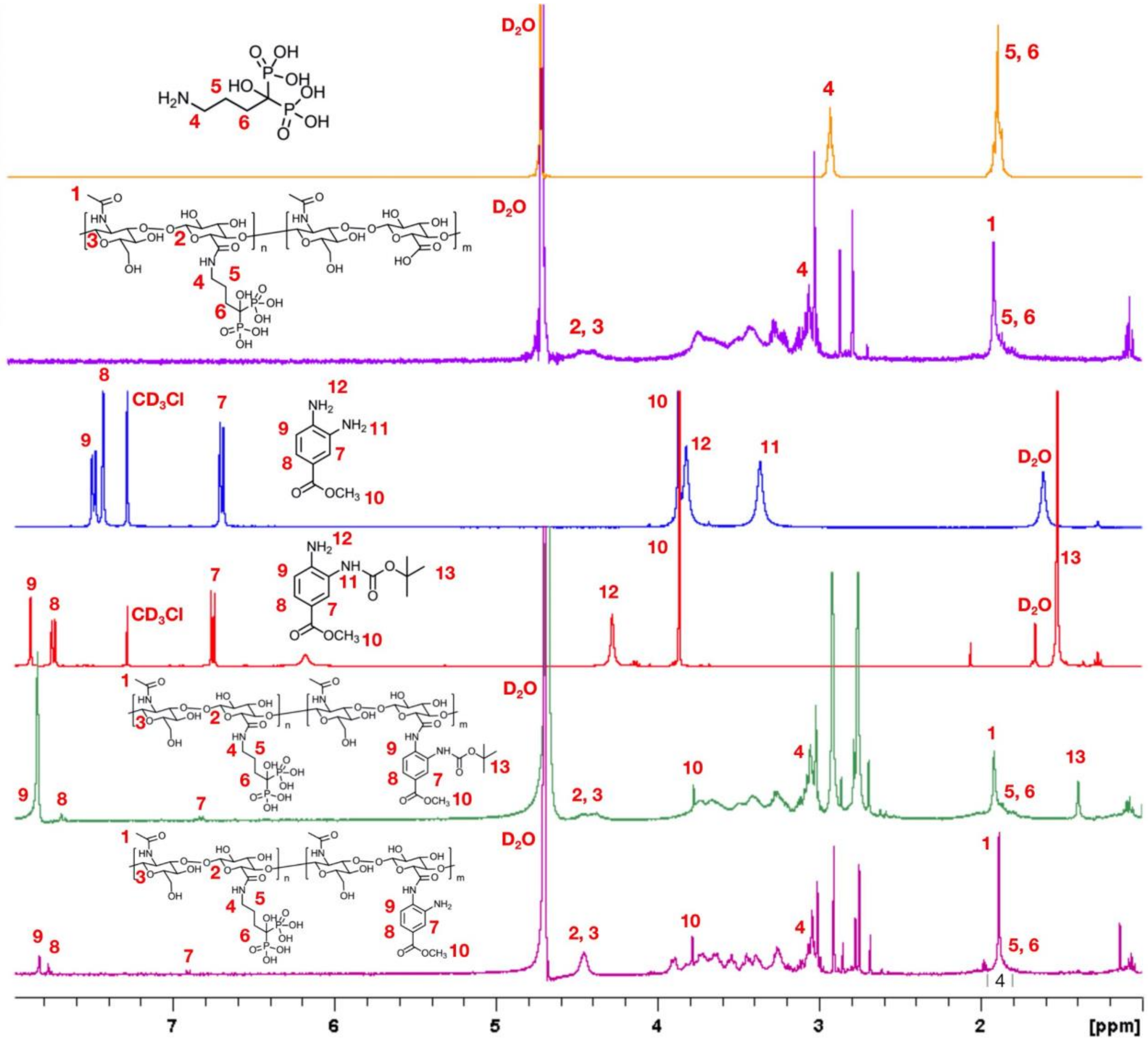

Figure S2. ^{1}H NMR spectrum of AHA and NAHA structures (400 MHz).

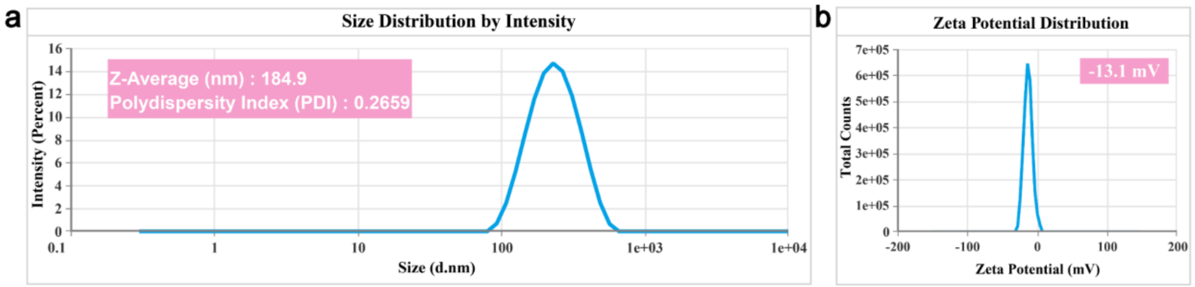

Figure S3. Size (a) and zeta potential (b) distribution of AHA-CaP/siRNA NPs detected using DLS.

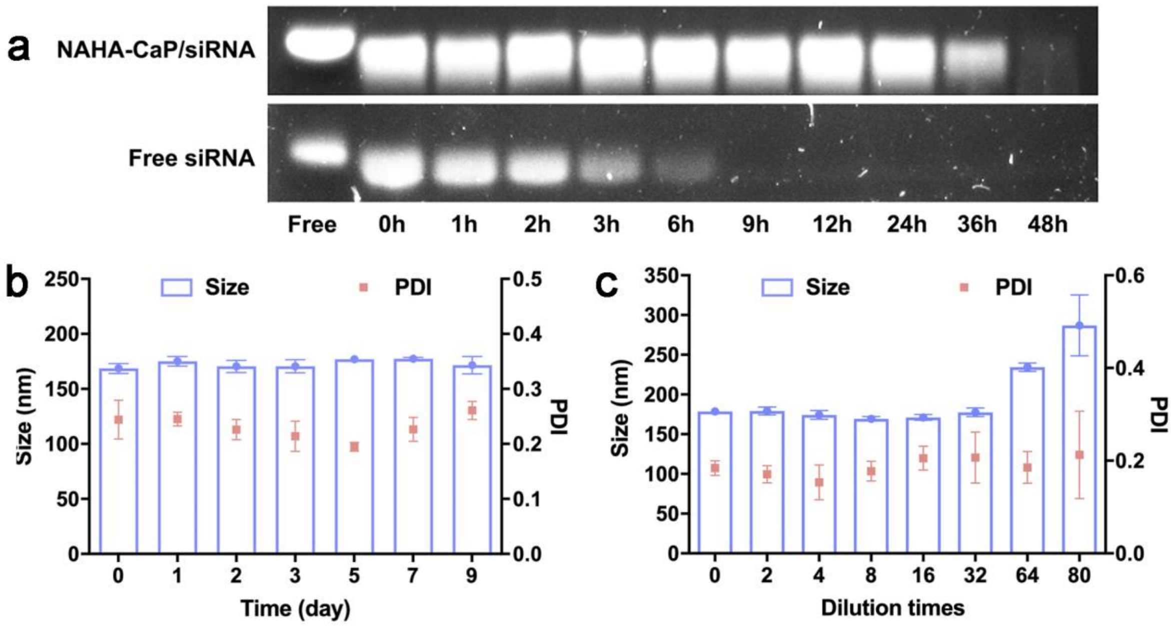

Figure S4. Characterization of NAHA-CaP/siRNA NPs. (a) The protective effects of NPs on siRNAs after incubation of NPs with FBS for different time detected using gel retardation assay. (b) Storage stability of NPs at 4℃ (n = 3). (c) Dilution stability of NPs in the HBS buffer (pH = 7.4) (n = 3).

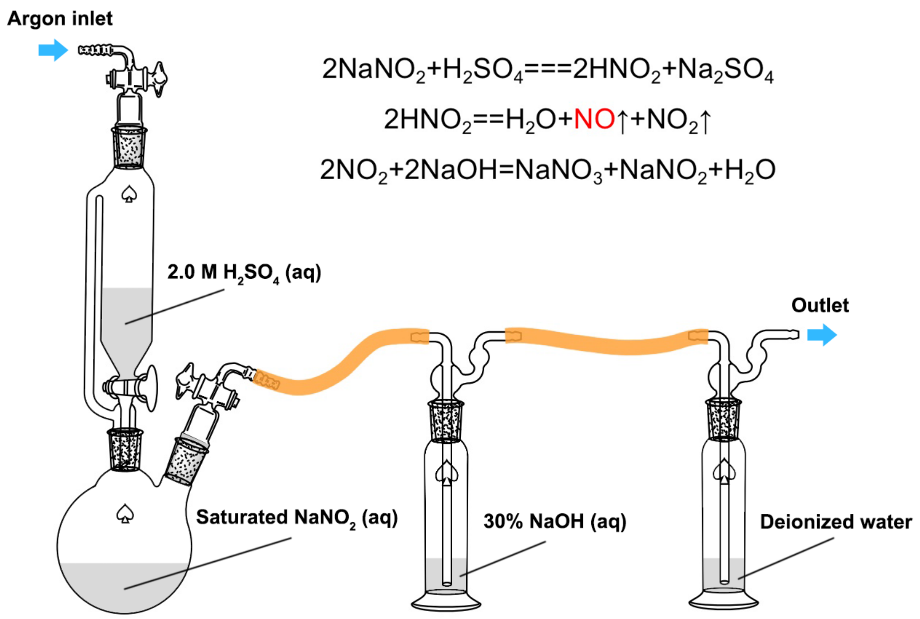

Figure S5. Scheme of the synthesis and collection device for NO gas.

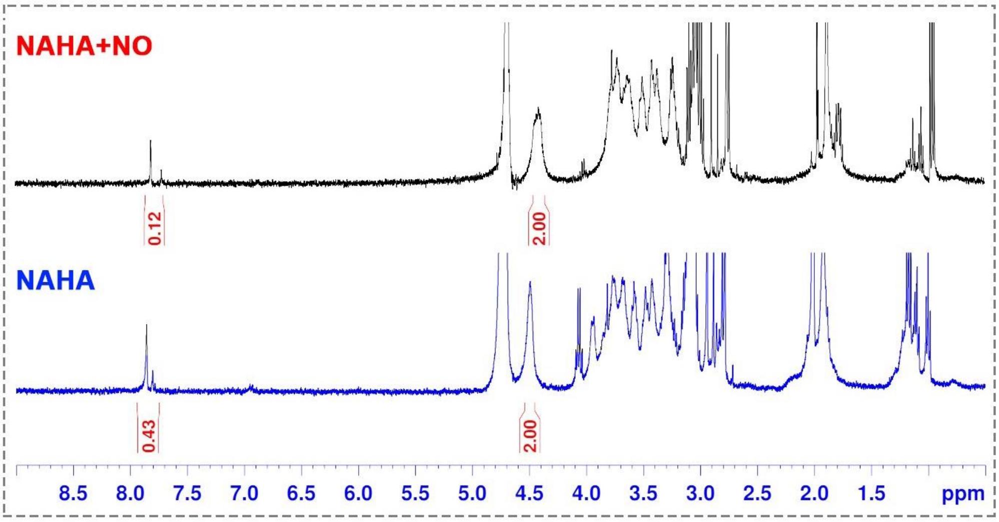

Figure S6. The ^{1}H NMR spectrum of NAHA material after treatment with or without NO.

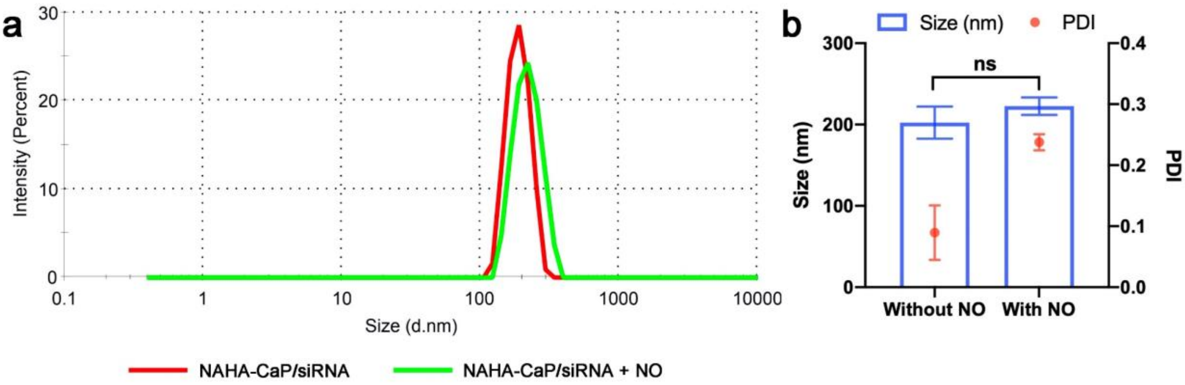

Figure S7. The changes in particle size of NAHA-CaP/siRNA NPs with or without NO. The data were shown as mean ± SD (n = 3). *$p < 0.05$, **$p < 0.01$, ***$p < 0.001$.

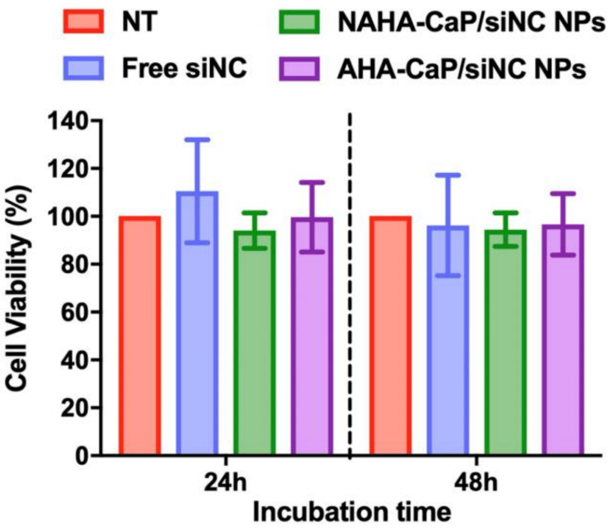

Figure S8. Cell viability of RAW264.7 cells after incubation cells with different formulations for 24 and 48 h. “NT” referred to the normal RAW264.7 macrophages without LPS treatment. Data represented mean ± SD (n = 6).

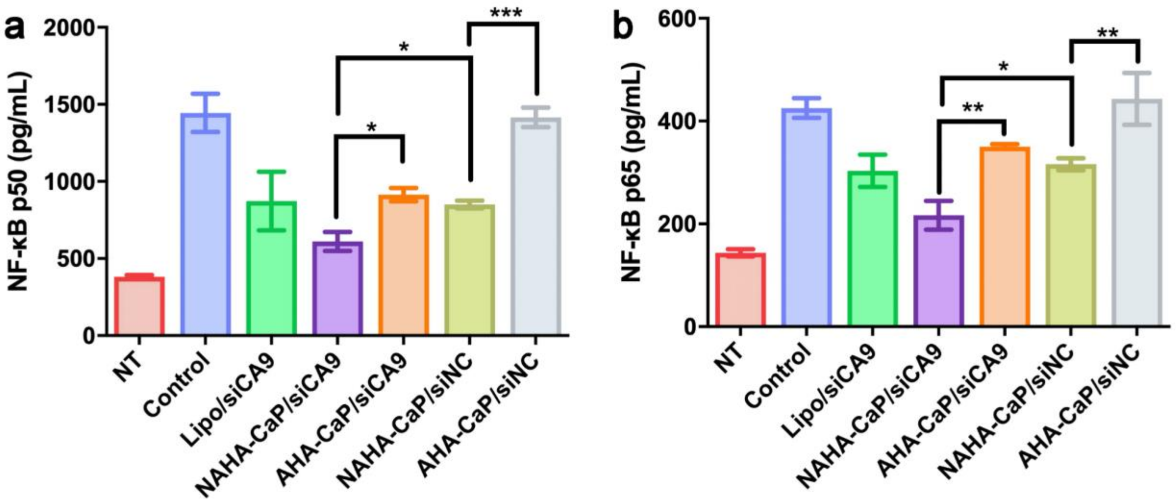

Figure S9. The concentrations of cytokines (a) NF-*κ*B p50 and (b) NF-*κ*B p65 in the LPS-activated RAW264.7 macrophages treated with different NPs detected using ELISA reagents. The data were shown as mean ± SD (n = 3). *$p < 0.05$, **$p < 0.01$, ***$p < 0.001$.

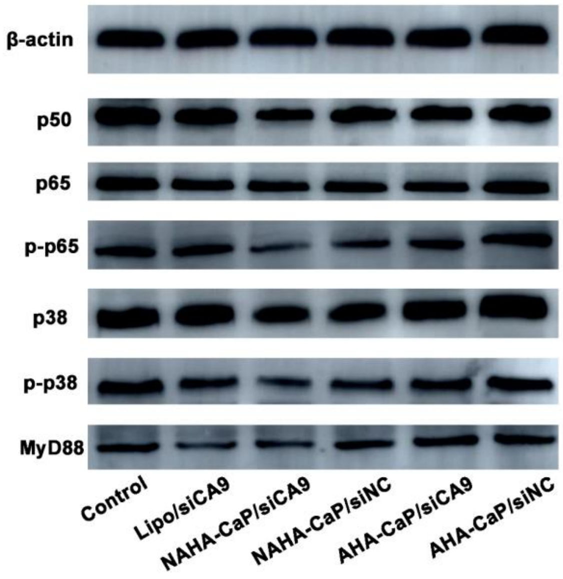

Figure S10. Western blotting images of inflammation-related protein expression in LPS-activated RAW264.7 macrophages after treatment cells with different NPs for 24 h.

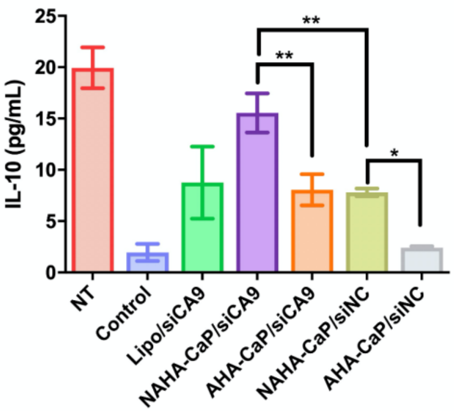

Figure S11. The intracellular concentration of IL-10 detected using ELISA after treating LPS-activated RAW264.7 cells with different NPs for 24 h. The data were shown as mean ± SD (n = 3). *p < 0.05, **p < 0.01, ***p < 0.001.

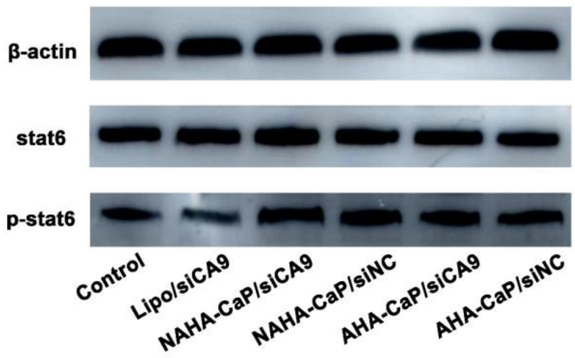

Figure S12. Western blotting images of STAT6 and p-STAT6 protein expression in LPS-activated RAW264.7 macrophages after treatment cells with different NPs for 24 h.

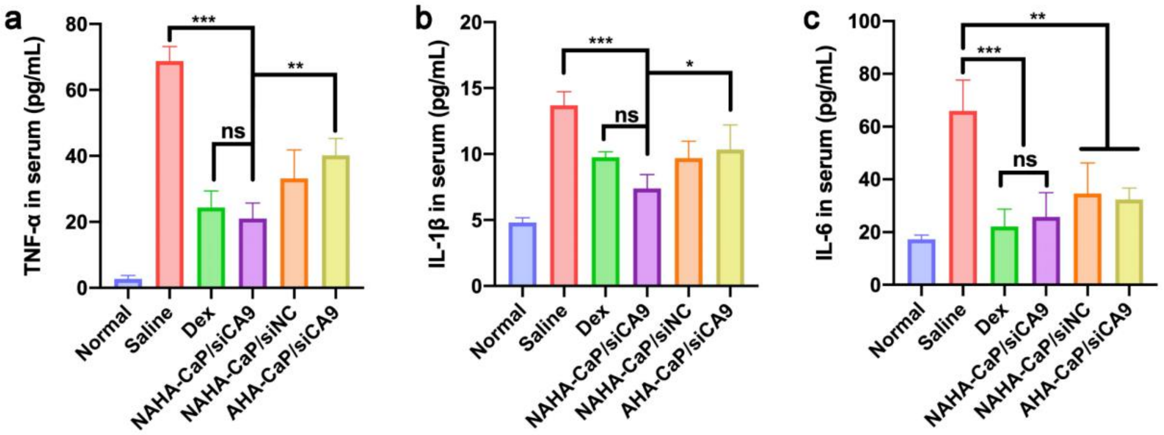

Figure S13. The concentrations of TNF-α, IL-1β, and IL-6 in serum from the mice with early-stage OA after treatment of different NPs (n = 3). Data were presented as the mean ± SD. *p < 0.05, **p < 0.01, ***p < 0.001.

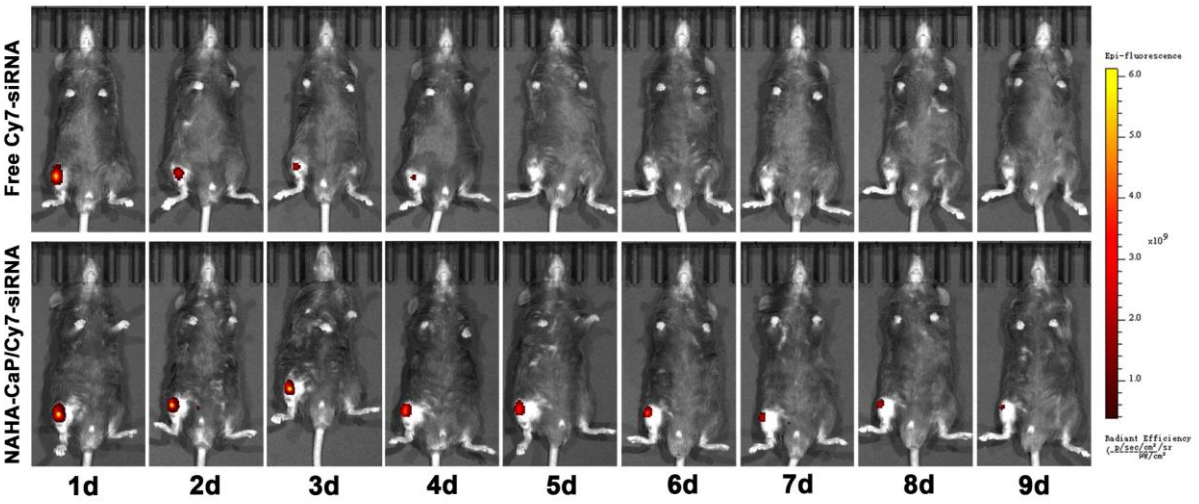

Figure S14. Representative IVIS images of knee joints from OA mice (OA was induced by MIA for eight weeks) after single intra-articular injection of free Cy7-siRNA or Cy7-siRNA loaded NAHA-CaP/siRNA NPs.

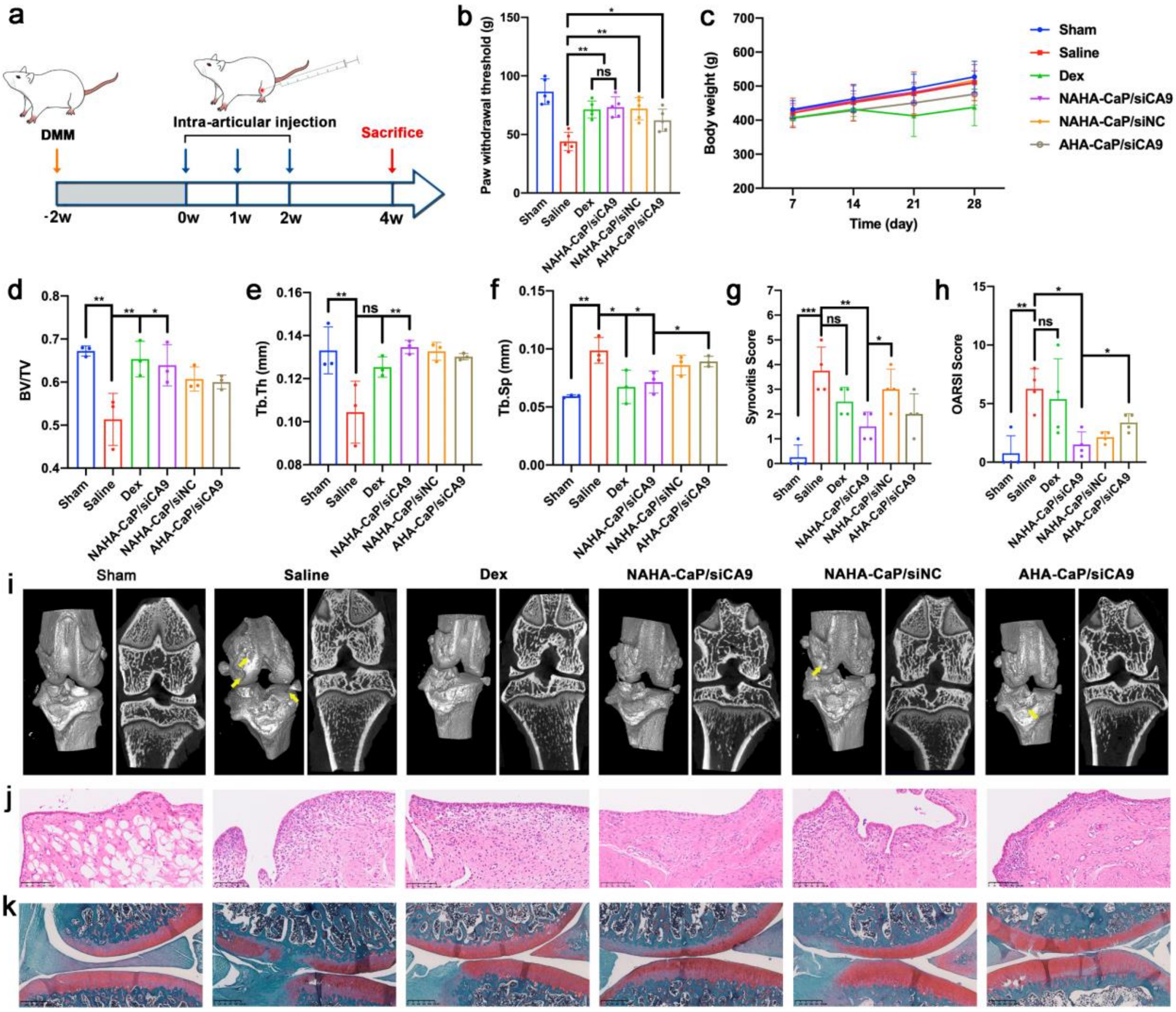

Figure S15. In vivo therapeutic effects of NPs in DMM-induced OA rat models. (a) Schematic illustration of the experimental design in SD rats with OA. (b) The paw withdrawal threshold evaluated using electronic von Frey aesthesiometer at the end of treatment. (n = 5). (c) The changes in body weight of rats with OA during entire treatment period (n = 5). (d) The quantitative measurements of Bone Volume Fraction (BV/TV), (e) Trabecular Thickness (Tb. Th), and (f) Trabecular Separation (Tb. Sp) detected using microCT (n = 3). (g) Synovitis score of H&E-stained sections (n = 4). (h) Cartilage OARSI score in Safranin O-fast green-stained sections (n = 4). (i) The representative microCT bone remodeling 3D and microCT 2D images of the knee joint tissues. (j) H&E staining images of knee joint tissues. Scale bars were 100 μm. (k) Safranin O-fast green staining images of knee joint tissues. Scale bars were 500 μm. The data were shown as mean ± SD. **p* < 0.05, ***p* < 0.01, ****p* < 0.001.

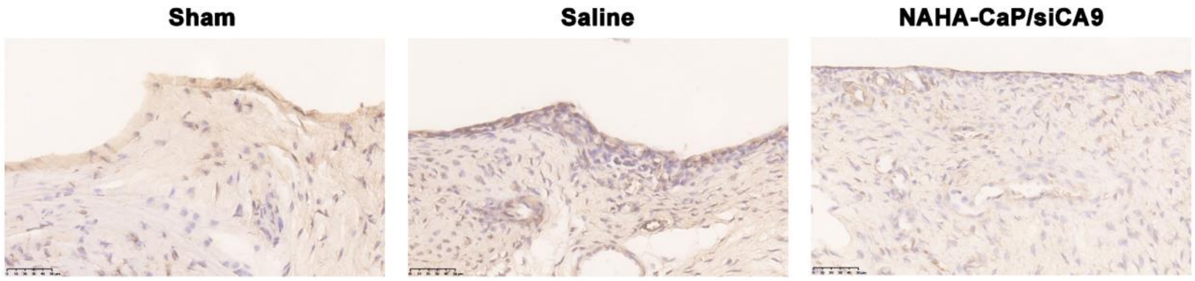

Figure S16. The expression of CA9 protein in the joint tissues isolated from the DMM-induced OA rat models detected using immunohistochemistry. Scale bars were 50 μm.

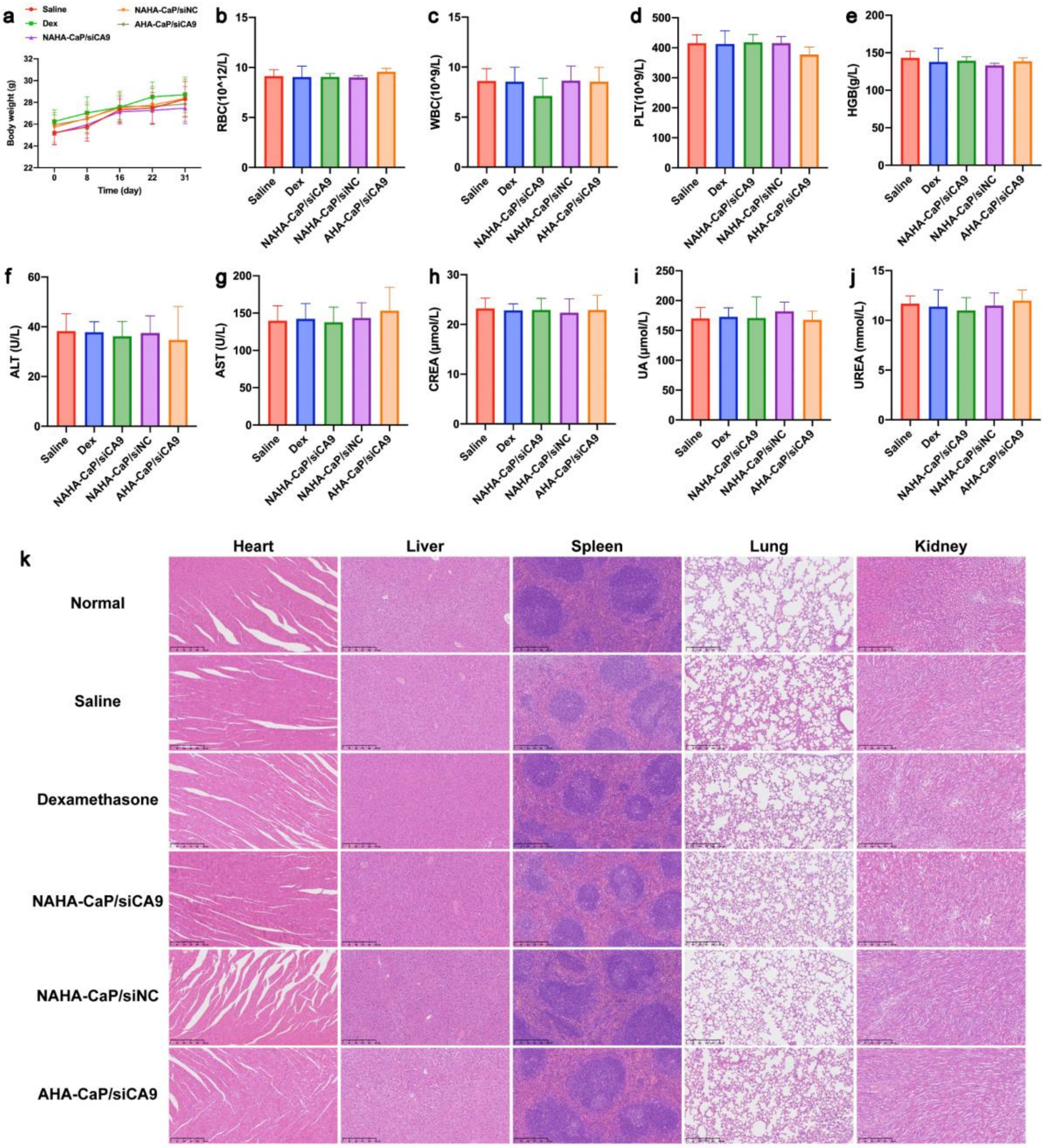

Figure S17. The biosafety of NPs in the mice with early-stage OA. (a) The changes of body weight of mice during the whole treatment period (n = 11). The levels of (b) RBC, (c) WBC, (d) PLT, and (e) HGB in the serum of OA model mice at the end of the treatment (n = 5). The levels of (f) ALT, (g) AST, (h) CREA, (i) UA and (j) UREA in the serum of OA model mice at the end of the treatment (n = 5). Data were presented as the mean ± SD. (k) Hematoxylin-eosin staining images of major organs from the mice after treatment with different NPs.

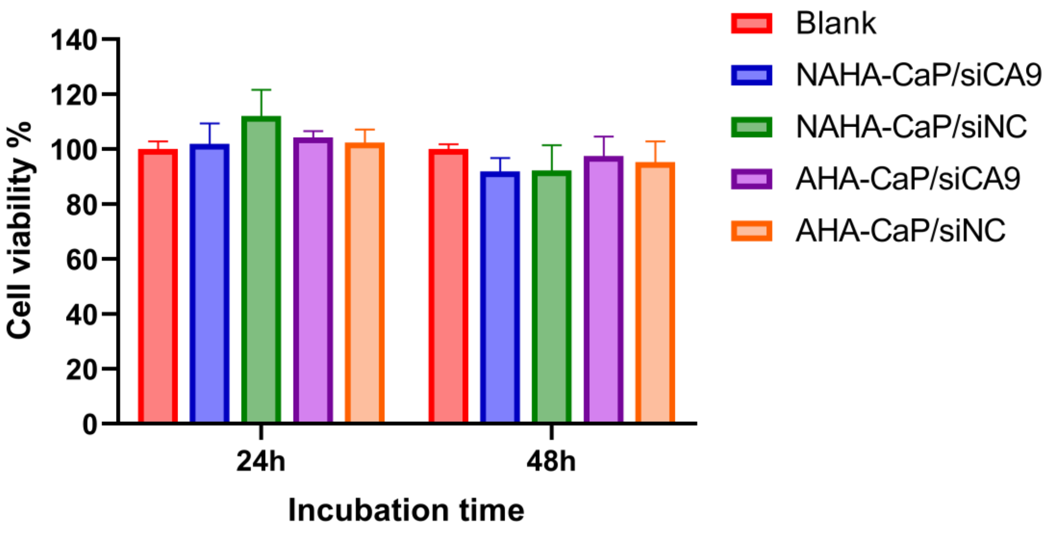

Figure S18. Cell viability of primary chondrocytes of mice after incubation cells with different formulations for 24 and 48 h. Data represented mean ± SD (n = 6).
